# Feasibility study of a co-designed, evidence-informed and community-based incentive intervention to promote healthy weight and well-being in disadvantaged communities in Scotland

**DOI:** 10.1136/bmjopen-2024-092908

**Published:** 2025-02-20

**Authors:** Julie Cowie, Scott Findlay, Rhonda Archibald, Sinead Currie, Pauline Campbell, Danielle Hutcheon, Marjon van der Pol, Graeme MacLennan, Elizabeth Cook, Bette Lock, Pat Hoddinott

**Affiliations:** 1 Glasgow Caledonian University, Glasgow, UK; 2 NHS Forth Valley, Stirling, UK; 3 University of Stirling, Stirling, UK; 4 University of Aberdeen, Aberdeen, UK; 5 Westfield Park Community Centre, Westfield, UK; 6 The Hope Hub, Denny, UK

**Keywords:** Community-Based Participatory Research, PUBLIC HEALTH, Overweight

## Abstract

**Abstract:**

**Objectives:**

To feasibility test a novel community-based financial incentive scheme to promote healthy weight and well-being.

**Design:**

Single-arm, prospective feasibility study using mixed methods.

**Setting:**

Two communities in Scotland experiencing high levels of disadvantage according to the Scottish Index for Multiple Deprivation (SIMD). Community C1 is in a large rural area with a small town centre (population~1.5K) and community C2 is a small and urban community (population~9K), enabling contextual comparison.

**Participants:**

Eligible adult (18 years or over) community members recruited through community outreach.

**Intervention:**

The Enjoy Life LocallY (ELLY) intervention comprised free soup twice weekly (café/delivery/pickup); loyalty card stamped for engagement in community assets (such as local activities, groups and clubs) exchanged for a £25 shopping card when a participant attends a minimum of 9 assets over 12 weeks; goal setting; information resources; self-monitoring of weight and well-being.

**Outcomes:**

Primary outcomes—feasibility of recruitment, retention and engagement. Acceptability of intervention components was assessed by self-reported questionnaires and interviews. Secondary outcomes—feasibility of collecting outcomes prioritised by communities for a future trial: health-related quality of life (EQ-5D-5L), mental well-being (WEMWBS), connectedness (Social Connectedness Scale) and weight-related measures (weight, body mass index (BMI)).

**Results:**

Over 3 months, 75 community citizens (35 citizens in C1, 40 citizens in C2) were recruited (125% of target recruitment of 60 participants (117% of 30 participants C1 target, 133% of 30 participants C2 target), 84% female, baseline weight mean (SD)=84.8 kg (20) and BMI mean (SD)=31.9 kg/m^2^ (7.3), 65/75 (87%) living in disadvantaged areas (SIMD quintiles 1–3)). Retention at 12 weeks, defined by completion of outcome measures at 12 weeks, was 65 (87%). Participation in at least one asset for a minimum of 9 out of 12 weeks of the intervention was achieved by 55 (73%). All intervention components were acceptable, with the loyalty card being the most popular and the soup cafés the least popular. The mean average cost of the soup ingredients, per participant, over the 12 weeks was £12.02. Outcome data showed a small decrease in weight and BMI and a small increase in health-related quality of life, mental well-being and social connectedness.

**Conclusions:**

The ELLY study recruited and retained participants from two disadvantaged communities in Scotland. The study was acceptable to participants and feasible to deliver. A full trial is warranted to determine effectiveness and cost-effectiveness, with consideration of scalability.

**Trial registration number:**

The ELLY feasibility study was not pre-registered.

**STRENGTHS AND LIMITATIONS OF THIS STUDY**

- The Enjoy Life LocallY intervention was co-designed with community citizens using a community-based participatory research approach.
- The study recruited across two disadvantaged communities to an asset-based, incentive, community intervention.
- The feasibility study was not powered to detect effects on weight-related or well-being outcomes and change in outcome measures should be interpreted with caution.
- Effectiveness of intervention components will need to be established in a future, larger-scale trial.

## Introduction

 People living in disadvantaged areas have poorer health and are dying younger through increased risk of obesity-related conditions including diabetes, heart disease, some cancers, and infections.^1^ The personal, NHS resource and societal costs of obesity are considerable.^2^ Multiple behaviours are obesity risk factors (eg, over-consuming high fat, high sugar food and drinks, physical inactivity) and these behaviours cluster within disadvantaged families and communities with adverse consequences throughout the life course.^3^

Solutions to support people living well can benefit from coproduction and involving people with lived experience, promoting equity and opportunity. There is a strong rationale for ‘putting the public back into public health’ through community-based action research working ‘with’ rather than imposing ideas ‘on’ communities.^4^

### Social prescribing and community assets approach

The accessibility and sustainability potential of the social prescribing approach, where citizens are connected to community resources to support their health and well-being needs, is an important consideration for community-based interventions.^5^ Systematic review evidence on the use of social prescribing to supporting disadvantaged communities has shown the approach to be effective in providing vulnerable groups with a means of bridging the gap between psychosocial support and medical services.^5 6^ The approach allows primary care to link/signpost patients to community assets/services and is effective in reducing noncommunicable diseases (eg, anxiety and depression)^7 8^ as well as reducing pressure on healthcare services.^9^ In addition, evidence is emerging on how building social resilience and cohesion within disadvantaged communities has an impact on health outcomes.^10^ Research that seeks to better understand the links between ‘social and community networks’ without a primary care gatekeeping role is important. In particular, community asset-based approaches to health improvement which are coproduced locally to be relevant to local circumstance and culture and where behaviours are studied in context show promise.^11 12^ Although there is consensus that such asset-based approaches show potential in supporting community health, the evidence base is limited.^13^,^15^ Community engagement can facilitate positive change on healthy behaviours and consequences; however, systematic review evidence shows that community interventions can generate health inequalities, as they engage more advantaged, time-rich and organised people.^16 17^

### Financial incentive interventions

Financial incentive interventions, when combined with effective behaviour change and engagement techniques, have the potential to prevent noncommunicable diseases,^18^,^20^ and engage people living in disadvantaged areas.^21^ Financial incentives offered to individuals show evidence of effectiveness for weight loss; however, there is a risk of weight regain once the incentive intervention is withdrawn.^22^ Evidence is limited for financial incentives delivered at a community level. Neighbourhood interventions to promote healthy weight are recommended in a recent UK biobank study, particularly for people at higher genetic risk of obesity.^23^ By targeting communities rather than individuals, there are opportunities for minimising weight stigma, which a meta-analysis of systematic reviews found has adverse psychological consequences, such as depression and anxiety.^24^

### Research aims

The aim of the study was to feasibility test a novel evidence-informed and community-based financial incentive intervention to promote healthy weight and well-being. Specifically, we assessed (1) the feasibility of recruiting participants from community venues and pop-up café events; (2) retention and engagement rates, acceptability of the intervention components, feasibility of delivery, fidelity and unintended consequences; (3) the feasibility of collecting outcome measures prioritised by communities: weight, well-being, health-related quality of life, social connectedness; and (4) effects on weight-related outcomes and well-being and progression criteria for a future large-scale evaluation.

## Methods

The Consolidated Standards of Reporting Trials extension for reporting feasibility and pilot trials was followed (see online supplemental file A).^25^

### Study design

The study design was a single-arm, prospective intervention feasibility study, using mixed methods to collect descriptive quantitative and qualitative data from community participants.

### Public and patient involvement

**Figure 1:**
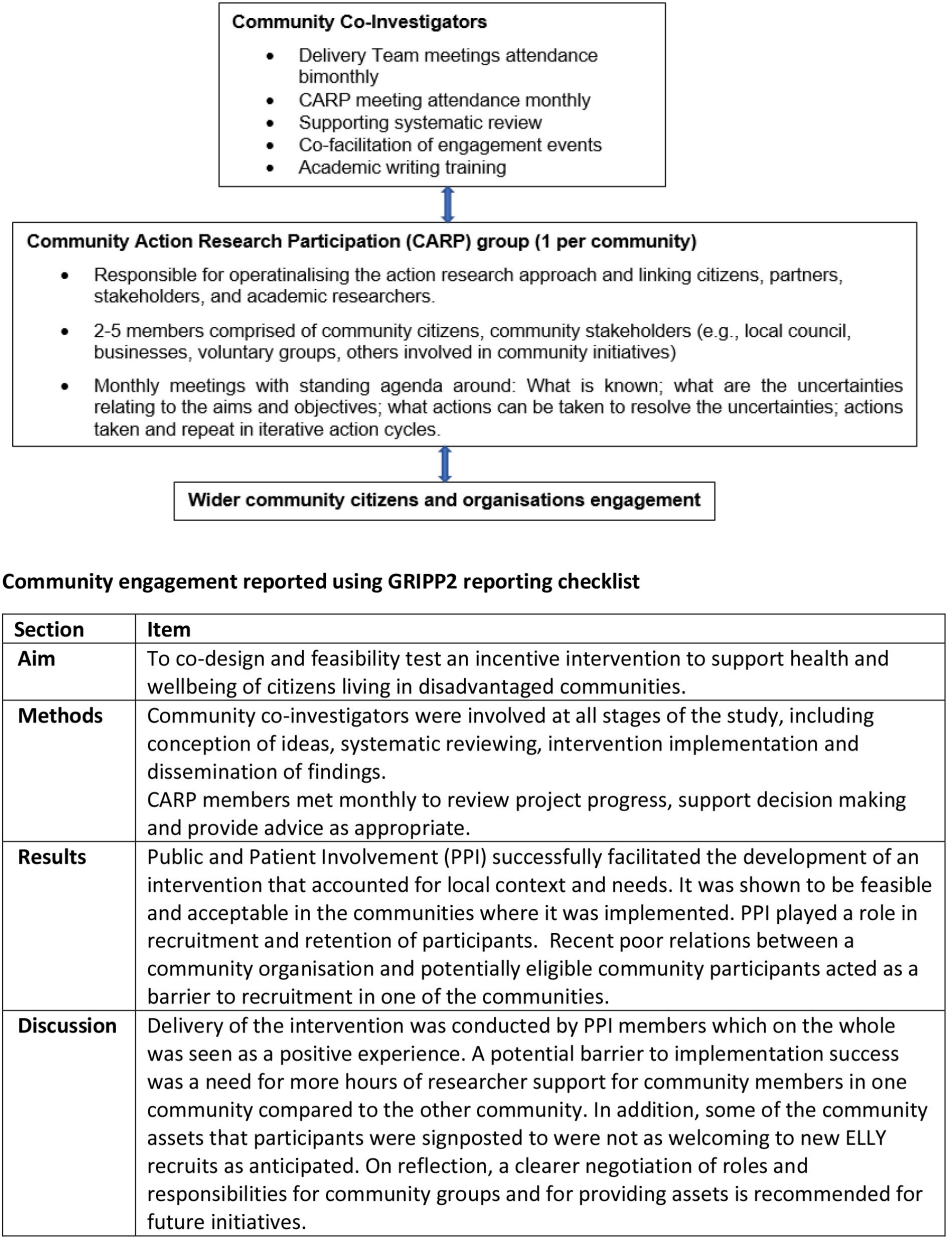
Community engagement in the Enjoy Life LocallY (ELLY) project. GRIPP2, Guidance for Reporting Involvement of Patients and the Public.

Public and patient involvement (PPI) was continuous and responsive, as described by Gamble *et al*.^26^ Community members participated in the project across four levels: as grant holder coapplicants, as members of Community Action Research Participation (CARP) groups and as volunteers. Community coinvestigators were instrumental in promoting the study, assisting with recruitment and cofacilitating community engagement events. Each CARP group (one per community) was responsible for operationalising the intervention and linking citizens, partners, stakeholders and researchers. A standing agenda at CARP meetings was as follows: what is known; what are the uncertainties relating to the aims and objectives; what actions can be taken to resolve the uncertainties; and actions taken. Figure 1 presents PPI roles and responsibilities, and PPI is described using the GRIPP2 (Guidance for Reporting Involvement of Patients and the Public) reporting guidance.^27^

### Setting

The academic team was approached by NHS Forth Valley Public Health Nutrition Team as healthy weight was a concern raised by citizens through the Local Authority Community Planning Process across disadvantaged communities in the region. Two disadvantaged communities (Scottish Index for Multiple Deprivation (SIMD) 1–3 (quintile)) in Forth Valley were chosen that were disparate in nature but felt representative of communities across the region and more widely, across Scotland. Researchers had no engagement with either community prior to the study commencing.

Housing in both communities predominantly comprised of public (social) housing. Assets in both communities are local activities, groups and clubs focusing on arts and crafts, physical activity, nutrition and socialising. Community (C1) is a small rural town, with population of approximately 8000 people. SIMD levels range from 1 to 3 (quintile) in the target area, with more affluent areas (SIMD 4–5) on the periphery. The community partners operated on two separate sides of the town and had no prior interactions. Local assets are based predominately at community hubs, the local library and church. The largest supermarket is a 10 min walk from the town centre with the alternative being local shops. Community 2 (C2) is a small and urban community, with population of approximately 9000 people. SIMD levels range from 1 to 2 (quintile). Local assets are mainly based at the community centre operated by our community partner. A retail park (and the closest supermarket) is a 20 min walk away with a small grocery shop and petrol station located in the target area.

### Eligibility criteria

Inclusion criteria: any adult (aged 18 or over) living within 20 min walking distance from main community assets were eligible to attend. Exclusion criteria: inability to understand project information, the commitment required and consent; not planning to reside in community for the duration of the intervention period.

### Participant recruitment

A wide range of recruitment methods were employed involving community groups, local business, pop-up cafes and school flyers. Equality of inclusion to ensure representativeness from all in the communities that might benefit from participating in the ELLY intervention was promoted through social media publicity, local adverts and door-to-door flyers. Community champions were identified to support recruitment. Recruitment took place June 2023 to August 2023. A weekly review of recruitment numbers was conducted and feedback from community citizens on methods used was acted on with new strategies (eg, researcher attending community groups, pop-ups at strategic locations) introduced as necessary. Community citizens were invited to express interest in study participation at events when an ELLY researcher was in attendance, at pop-up cafes or by contacting the research team via email/phone/text/ELLY website.

### Baseline appointment

Having expressed interest, participants received a participant information sheet and were invited to attend a baseline appointment with a researcher at a date/time and location of their choice (eg, home, community centre, library). At the baseline, appointment participants (i) were assessed for eligibility; (ii) provided written consent to take part; (iii) self-completed baseline questionnaires; (iv) allowed the researcher to take their height and weight measurements and; (v) set weight, well-being and/or personal goals. The topic of goal setting (rationale and how it can be helpful) had already been introduced to participants in the ELLY Participant Information Sheet. In the baseline appointment, the researcher and participant engaged in discussion around potential goals the participant may wish to set. The mean average time taken for baseline appointments was 45 min, with questionnaire completion taking an average 20 min, and goal setting discussions, taking an average of 10 min.

### Intervention components

**Figure 2:**
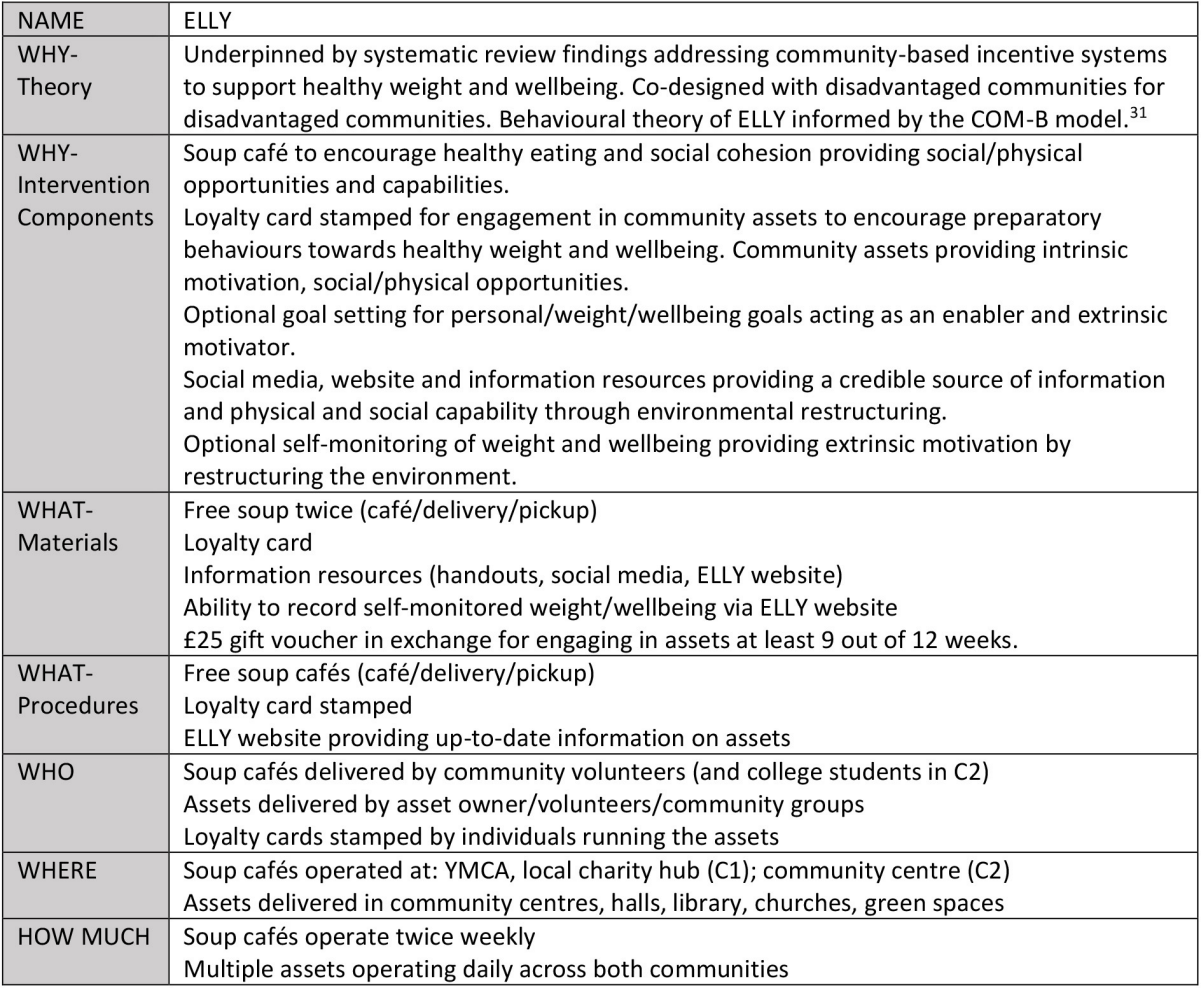
Template for Intervention Description and Replication checklist for the Enjoy Life LocallY (ELLY) intervention.

The ELLY study adopted a community-based participatory research approach,^28^ where community members were active and engaged at all stages of the research process. It was co-designed by two disadvantaged communities for use in disadvantaged communities. Development of the ELLY intervention was informed by guidance on development and evaluation of complex interventions (MRC/UKRI Guidance on complex interventions).^29^ The framework by Adams *et al*^30^ was used to identify all domains of the incentive scheme for which choices needed to be made. The behavioural theory of ELLY was informed by the COM-B model.^31^ The intervention is described using the Template for Intervention Description and Replication checklist,^32^ a summary of which is provided in figure 2.

The ELLY intervention is a place-based, asset-based incentive system. Community consultation indicated that an intervention focusing solely on weight was felt stigmatising and not inclusive of all community citizens. Citizens expressed a desire for an intervention to support them as a ‘whole person’ (recognising mental, physical, social, spiritual aspects), rather than a focus on one component alone. The resulting intervention adopts a holistic approach to supporting healthy weight and well-being, acting as a connector to existing assets and promoting autonomy. The intervention is not prescriptive in which ELLY assets participants should engage in, or exclusive in incorporating only assets seen to be directly supportive of healthy weight and/or well-being (eg, a walking club). Assets such as a writing group or craft club (two ELLY assets in C2), which may have indirect benefits to healthy weight and well-being, such as providing friendship, reducing social isolation and providing an opportunity for physical activity, were included.

The intervention places significant emphasis on social cohesion, connectedness and relationships and the role these play on supporting individuals to live well. The ELLY intervention builds on learning from previous studies the authors have undertaken, particularly around financial incentive design, preparatory behaviours, successful community recruitment and signposting to support resources.^33 34^ The intervention includes elements to motivate preparatory behaviours towards weight-related and well-being outcomes and promote commitment, and has embedded tailored evidence-based behaviour change techniques (goal setting, social support, demonstration of behaviour, adding objects to/restructuring the environment). An ELLY theory of change model^35^ was developed describing intervention components and function, behaviour change taxonomy elements addressed, and perceived outcomes (immediate, intermediate and long term).^31^ (see online supplemental file B for ELLY theory of change model).

ELLY is a 12-week intervention comprising of (1) provision of free soup twice weekly (café/delivery to home/pickup) for all participants; (2) a loyalty card stamped for engagement in local assets to encourage preparatory behaviours towards key outcomes (weight-related/well-being/social connectedness). Assets include local activities, groups and clubs in the community that agreed to be part of the ELLY intervention. Assets are broad and inclusive (informed by community consultation) comprising of arts and crafts, physical activity, nutrition-related, and social groups. Assets were usually free to attend, with only 1/22 activities and 6/24 charging a small fee in communities C1 and C2, respectively (see online supplemental file C for a full list of assets eligible for the loyalty card incentive in each community). Participants who achieved nine stamps on their loyalty card (equating to attending at least one activity, per week, over 9 out of 12 weeks of the ELLY intervention) were rewarded with a £25 shopping card at 12 weeks; (3) the option to set goals. Goal setting options were discussed at the baseline appointment, where participants were informed about the optional aspect of goal setting for ‘living well’. Participants were given the opportunity to set (outcome or behaviour) goals under the topics of personal, weight and well-being. Goal setting was participant driven; however, the researcher encouraged generation of SMART (Specific, Measurable, Achievable, Relevant, and Time-bound) goals to achieve over the 12 weeks. No specific action plans were developed; however, the researcher signposted the participants to the other intervention components and community assets. Goals set were reviewed at 12-week appointments; (4) website/written materials with access to local asset/activity ‘What’s on’ information and optional self-monitoring of weight and well-being via the website.

#### Outcomes

Table 1 summarises the outcomes, measures/approaches, data source and analyses corresponding to the study objectives.

**Table 1:** Study outcomes, measures/approaches, data source and analyses corresponding to the study objectives

Target	Objective	Measure/approaches	Data source	Analysis
Recruitment	Feasibility of recruiting 60 participants (30 per community) within 3 months	Recruitment rateRecruitment activitiesRecruitment timelineParticipant interviewsResearchers’ field notes	Recruitment informationInterview transcriptsField notes	Descriptive statisticsThematic qualitative analysis
Retention	Attendance for 12w outcome measuresNumber of participants receiving voucher for attendance	QuestionnairesWeight measurementsNumber of withdrawals, 12w data collection	ELLY questionnaires: The Warwick-Edinburgh Mental Wellbeing Scales (WEMWBS),^38^ EQ-5D-5L,^39^ the Social Connectedness Scale – Revised,^40^ Social connectedness, and ELLY-specific questionnairesDiary of communicationHeight/weight measurements	Descriptive statistics
Intervention	Acceptability and feasibility of intervention components	QuestionnairesInterviewsAccess to intervention components	ELLY questionnairesInterview transcriptsField notesLoyalty card stamps	Descriptive statisticsThematic qualitative analysis
Fidelity and un-intended consequences	Delivery of the intervention components or study procedures as intended. Unintended consequences	InterviewsQuestionnairesField notes	ELLY questionnairesInterview transcriptsField notesDiary of communication	Descriptive statisticsThematic qualitative analysis
Outcome measures	Feasibility of collection	QuestionnairesWeight measures	Validated (EQ-5D-5L, WEMWBS, Social connectedness)Weight measures	Descriptive statistics
Effect observed	Change in well-being, weight-related outcomes, engagement at 12 weeks	QuestionnairesWeight measuresInterviews	Validated (EQ-5D-5L, WEMWBS, Social connectedness) and ELLY-specific questionnairesInterview transcriptsWeight measuresGoal setting data	Descriptive statistics

ELLYEnjoy Life LocallY

An independent study steering group, comprised of both academic experts and lay members, advised whether the following prespecified progression criteria were sufficiently met to proceed to a full trial:

1. Acceptability of the intervention and individual components by the majority of participants.
2. Feasibility of recruiting at least 30 citizens in each community in 3 months.
3. 12-week outcomes collected from 75% of participants based on Macaulay *et al.*^36^
4. Evidence of indicative effects on outcomes collected.

#### Outcome assessment

Outcomes were assessed at baseline and 12 weeks (at end of intervention). Individual appointments were conducted by a researcher at community centres, the local college (C2) and participants’ homes, depending on participant preference. Travel expenses were not provided.

Height was measured at baseline using a portable stadiometer to the nearest 0.1 cm. Weight was measured at baseline and 12 weeks. Prior to weight measurement participants removed shoes and bulky clothing. Weight was recorded using portable calibrated scales to the nearest 0.01 kg. The Scottish health survey^37^ was used to provide BMI categories. Information on adverse events was recorded at assessments or at the time of reporting if during the 12-week intervention. Adverse events related to participants becoming unwell or distressed, or disclosing information relating to a health condition during the study.

The self-reported questionnaires used for collection of outcome data were informed by community consultation and the ELLY intervention theory of change model. Validated questionnaires were used to capture well-being (The Warwick-Edinburgh Mental Wellbeing Scales (WEMWBS))^38^ and quality of life (EQ-5D-5L).^39^ Existing and adapted questionnaires were used to capture responses relating to social connectedness,^40^ socio-demographics, comorbidities, disabilities,^41^ lifestyle choices^42^,^45^ and interaction with NHS services.^37^ Questionnaires were completed during the appointment with a researcher (baseline) and at home online prior to/during appointments (12 weeks).

Participants’ engagement with and experience of the ELLY intervention components were assessed using an ELLY 12-week questionnaire (see online supplemental file D). Specifically:

- Engagement with ELLY activities was assessed by asking participants to ‘Please indicate (with a tick) how often you attend the following types of activities in the last 12 weeks?’ for each category of ‘Arts & crafts activity’, ‘Physical Activity group’, ‘Nutrition group’, Social related group’ and ‘Other (please specify)’. Response options were ‘0–1 over 12 weeks’, ‘2–4 over 12 weeks’, ‘3–5 over 12 weeks’ and ‘6+over 12 weeks’.
- Engagement with the ELLY soup provision was assessed by asking participants ‘If you took up the offer of soup twice a week, how did you get your soup? (please tick all that apply)’ with responses captured using the options of ‘Sit in at café, twice weekly’, ‘Collect soup twice weekly from café’, ‘Collect 2 portions of soup once a week from café’, ‘Delivered to house’ and ‘Other (please state)’.
- Acceptability of ELLY activities, loyalty card and reward, and soup provision was assessed by asking participants to ‘Please tick the box that best describes your experience of [‘attending local activities’/‘loyalty card and reward’/‘twice weekly free soup’] as part of the ELLY project’ followed by a series of statements, with responses captured using a Likert scale ranging from ‘Strongly disagree’ to ‘Strongly agree’ and ‘Not relevant’ provided as an option if participants did not feel the question was reflective of their experience. Free-text questions were also used to provide supplementary detail. General reflections on the ELLY intervention as a whole were captured using six open-ended questions at the end of the questionnaire.

Goal setting was conducted at baseline and goals reviewed at 12-week appointments. Goal setting and review were conducted using face-to-face interviews with participants. Personal goals were unrestricted and chosen by the participant. Weight goals allowed participants to ‘decrease’/‘stay same’/‘increase’ weight. Well-being goals were adapted from the EQ-5D descriptive system and VAS score.

At the 12-week appointment, all participants providing outcome data including weight (measured by a researcher) received a £25 shopping card as a thank you for their time. The website automatically recorded any self-reported weight entered by participants.

### Qualitative interviews

Participants were approached to take part in a semistructured interview at 12 weeks to gather qualitative data on their experiences of the ELLY study and related impacts on their health and/or well-being. Purposive sampling from both communities was informed by baseline participant characteristics and informal feedback from intervention volunteers relating to diversity of participants’ demographics, engagement and perspectives. Before commencing an interview, participants were provided with an information sheet and written consent form. Participants were also assured of their anonymity and right to withdraw at any point of the interview. Face-to-face interviews were conducted with participants at 12 weeks. All interviews followed a predefined topic guide (see online supplemental file E), lasted approximately 30 min and were audio recorded using an encrypted Dictaphone, then transferred to a password-encrypted computer folder. Researcher field notes were taken at all interviews and used to inform the qualitative analysis.

### Sample size

The study aimed to recruit 60 participants (30 at each community) to be sufficient in testing feasibility based on an estimated proportion of 5% for unforeseen problems (assuming a 95% confidence level).^46^

### Analysis

#### Quantitative analysis

Data from validated outcome questionnaires were analysed according to the guidelines provided by each measure. Participant characteristics and outcomes were summarised using descriptive statistics: mean (SD) for continuous variables and number (percent) for categorical variables. Likert scale variables were treated as continuous measures. The frequency, percentages and 95% CIs of observed levels are reported for all categorical variables. The proportion of individuals who expressed interest in the study, those recruited, retained and withdrawn at each stage, in each community was determined. CIs for proportions were calculated by the study statistician and derived using the normal approximation and for means using the standard normal distribution. Missing data were handled by following the appropriate guidelines for each scale, with the exception of the Social Connectedness Scale – Revised, where in the absence of guidelines, we applied an adaptation of the WEMWBS guidelines as used by Phillips *et al*.^47^ For weight-related outcomes, observed data only were included.

#### Qualitative analysis

NVivo V.12 software was used to support analysis of the qualitative data from interviews, free-text questionnaire responses and researcher field notes. Descriptive coding techniques were used to undertake initial thematic categorising of the data.^48^ A coding frame was developed independently by two researchers reading a diverse sample of five interviews, followed by team discussions to finalise the frame and identify key themes. Each theme was then explored in further detail and broken down into subthemes, with duplicate codes being merged. Analysis was performed by one researcher (DH), with 10% of data being cross-checked by a second researcher (RA), and regular review of coding and analysis by the PI (JC). Researcher field notes contributed to interview data interpretation. Emergent themes were discussed at weekly team meetings and at monthly CARP meetings. DH had not been involved in any other aspect of the research and her involvement in the qualitative coding added to the rigour and impartiality of the analysis.

## Results

**Figure 3:**
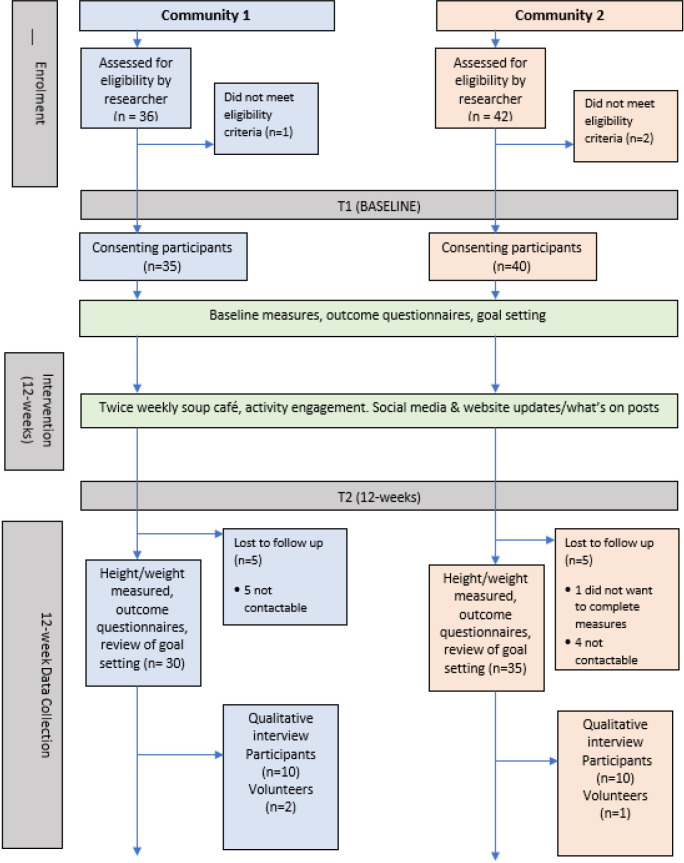
Consolidated Standards of Reporting Trials flow diagram of the Enjoy Life LocallY (ELLY) intervention.

Figure 3 depicts participant flow through each stage of the ELLY intervention.

### Recruitment and retention

Prior to the recruitment period, the research team held pop-up cafés in the communities and supported local events/activities with a view to increasing ELLY visibility between January and June 2023, becoming accepted faces in the community and promoting the research. Expressions of interest were made by 117 community citizens during this period. Recruitment was conducted over 3 months (June to August 2023), with 43 recruitment events/visits/pop-ups being held. Target recruitment of 30 per community was exceeded (C1—35 participants, C2—40 participants). The number of people recruited each week was on average three per community, with the majority (C1: 17/35 (49%), C2: 28/40 (70%)) of participants recruited from existing community groups/activities they attended. Recruitment via community partners and snowball recruitment were effective strategies in community C1 (9/35 (26%) and 5/35 (14%), respectively), and pop-up cafes/attending community events (eg, gala day) was an effective strategy in C2 (8/40 (20%)). Being weighed at baseline and 12 weeks was an initial barrier to one potential recruit. The importance of this outcome measure was discussed and reassurance around anonymity and use of data given, after which the individual was successfully recruited to the study.

Baseline appointments were attended by 78 citizens and 75 met required eligibility criteria. Those not eligible lived outside the target area (n=2) or planned to move away during the intervention (n=1). Questionnaire completion took time, and many participants expressed a preference for an online version and/or being able to complete the questionnaire at home, prior to the appointment. 20 participants agreed to be interviewed at 12 weeks.

Participation in at least one activity for a minimum of 9/12 weeks (assessed by 9 stamps on the 12-stamp loyalty card) and receipt of a £25 shopping card was achieved by 55/75 (73%) of study participants. Retention at 12 weeks, defined by completion of the 12-week outcome measures assessment, was completed by 65/75 participants (87%) with minimal difference in retention between communities (C1 30/35 (86%) retention, C2 35/40 (88%) retention). At 12 weeks, nine participants were lost to follow-up due to not being contactable and one participant withdrew from the study, as they did not wish to complete outcome measures. The proportion of drop-outs living in SIMD 1–3 was 8/10 (80%) which was reflective of the proportion of overall participants living in these SIMD categories. Of those contacted for interview at 12 weeks (10 per community), all agreed to be interviewed.

### Baseline characteristics

Table 2 reports the baseline characteristics of participants. Mean average age of participants was 53.3 (SD=16.7), with mean average weight of 84.8 kg (SD=20) and mean average BMI of 31.9 kg/m^2^ (SD=7.3). 63/75 (84%) of participants were female and 65 (87%) lived in disadvantaged areas (as defined by SIMD quintiles 1–3). Marital status was mixed, with married/civil partnership/cohabiting (32 participants (43%)) representing the largest classification group. Multiple long-term conditions were reported by 30 (40%) participants, 62 (83%) were ethnic group white and 12 (16%) were educated to degree level with 44 (59%) having some other form of qualification. The proportion of participants living alone was 23 (31%) and overall average household size was 2.6. Working status was mixed with retirees (25 (33%)) followed by those not in paid working due to long-term illness/disability/other reason (20 (27%)) representing the largest classification groups.

**Table 2:** Participant baseline characteristics

	C1 n=35	C2 n=40	Total n=75
Age (years), mean (SD)	56.5 (18)	50.4 (15)	53.3 (17)
Gender, n (%)			
Female	29 (83)	34 (85)	63 (84)
Male	6 (17)	6 (15)	12 (16)
Height (cm), mean (SD)	162.1 (9)	163.9 (7)	163 (8)
Weight (kg), mean (SD)	83.9 (17)	85.6 (23)	84.8 (20)
BMI (kg/m^2^), mean (SD)	32.1 (7)	31.7 (8)	31.9 (7)
BMI (kg/m^2^), categories, n (%)			
Healthy weight (18.5≤BMI 18.5≤24.9)	5 (14)	7 (18)	12 (16)
Overweight (25.0≤BMI≤29.0)	10 (29)	6 (15)	16 (21)
Obesity/morbid obesity (30.0≤BMI)	20 (57)	26 (65)	46 (66)
Underweight (BMI<18.5)	0 (0)	1 (3)	1 (1)
SIMD deprivation category, n (%)			
SIMD 1 (most disadvantaged)	11 (31)	7 (18)	18 (24)
SIMD 2	10 (29)	20 (50)	30 (40)
SIMD 3	10 (29)	7 (18)	17 (23)
SIMD 4/5 (least disadvantaged)	4 (11)	6 (15)	10 (13)
Marital status, n (%)			
Married/civil partnership/cohabiting	15 (43)	17 (43)	32 (43)
Separated/widowed/divorced	9 (26)	13 (33)	22 (29)
Single (never married and never registered in a civil partnership)	10 (29)	8 (20)	18 (24)
Prefer not to say	1 (3)	2 (5)	3 (4)
Comorbidities, n (%)			
A stroke (including mini-stroke)	2 (6)	3 (8)	5 (7)
High blood pressure	12 (34)	10 (25)	22 (29)
A heart condition such as angina or atrial fibrillation	8 (23)	6 (15)	14 (19)
Diabetes	11 (31)	3 (8)	14 (19)
Cancer	3 (9)	4 (10)	7 (9)
Arthritis	9 (26)	12 (30)	21 (28)
A mental health condition	14 (40)	18 (45)	32 (43)
None of the above	10 (29)	14 (35)	24 (32)
Report a single comorbidity	9 (26)	12 (30)	21 (28)
Report multiple long term conditions	16 (46)	14 (35)	30 (40)
Ethnic group, n (%)			
Asian or Asian British	2 (6)	7 (18)	9 (12)
Black, African, Caribbean or Black British	0 (0)	1 (3)	1 (1)
Mixed or multiple ethnic groups	0 (0)	1 (3)	1 (1)
Other ethnic group	0 (0)	2 (5)	2 (3)
White	33 (94)	29 (73)	62 (83)
Education, n (%)			
At degree level or above	2 (6)	10 (25)	12 (16)
Another kind of qualification	21 (60)	23 (58)	44 (59)
Prefer not to say	2 (6)	1 (3)	3 (4)
No formal qualifications	6 (17)	3 (8)	9 (12)
Not reported	4 (11)	3 (8)	7 (10)
Household status			
Household size, mean (SD)	2.4 (1)	2.8 (2)	2.6 (2)
Living alone, n (%)	10 (29)	13 (33)	23 (31)
Working status, n (%)			
Have paid job—full time (30+ hours per week)	2 (6)	4 (10)	6 (8)
Have paid job—part time (29 hours or less)	1 (3)	7 (18)	8 (11)
Unemployed and seeking work	2 (6)	4 (10)	6 (8)
Retired	16 (46)	9 (23)	25 (33)
Full-time student	0 (0)	1 (3)	1 (1)
Not in paid work due to long term illness/disability/other reason	9 (26)	11 (28)	20 (27)
Not reported/other/prefer not to say	5 (14)	4 (10)	9 (12)

BMIbody mass indexSIMDScottish Index for Multiple Deprivation

### Acceptability of intervention components

For each intervention component, the survey responses are presented in table 3 followed by qualitative perspectives from 12-week participant interviews. Quotes have been chosen to represent the diversity of responses relating to the ELLY study in terms of engagement, acceptability and demographics of participants.

**Table 3:** Overall acceptability of the Enjoy Life LocallY (ELLY) components

ELLY components		C1 n=35	C2 n=40	Total n=75 (95% CI)
Soup n (%)				
Engaged in twice weekly soup (sit in/take away/delivery)		23 (66)	26 (65)	49 (65) (54, 76)
(Strongly agree/agree) getting soup was very convenient		16 (46)	17 (43)	33 (44) (33, 56)
(Strongly agree/agree) I made new friends as a result of ELLY soup		19 (54)	17 (43)	36 (48) (63, 60)
(Strongly agree/agree) ELLY soup helped me feel more part of my community		17 (49)	18 (45)	35 (47) (36, 59)
(Strongly agree/agree) ELLY soup kept me motivated		16 (46)	13 (33)	29 (39) (28, 51)
(Strongly agree/agree) ELLY soup was an important part of ELLY		18 (51)	17 (43)	35 (47) (36, 59)
(Strongly agree/agree) soup component helped with…	weight goal	7 (20)	8 (20)	15 (20) (12, 31)
	well-being goal	13 (37)	14 (35)	27 (36) (25, 48)
	personal goal	13 (37)	15 (38)	28 (37) (26, 49)
Community assets n (%)				
Participants engaged in at least one activity per week in at least 9 of the 12-week intervention		25 (71)	30 (75)	55 (73) (62, 83)
Participants engaged in more activities during the 12-week intervention than they did before		24 (69)	18 (45)	52 (69) (58, 79)
Participants attended new activities during the project		23 (66)	19 (48)	42 (56) (44, 67)
(Strongly agree/agree) I made new friends as a result of the activities		21 (60)	25 (63)	46 (61) (49, 72)
(Strongly agree/agree) the activities helped me feel more part of my community		24 (69)	25 (63)	49 (65) (53, 76)
(Strongly agree/agree) the activities kept me motivated		24 (69)	27 (68)	51 (68) (56, 78)
(Strongly agree/agree) the activities were an important part of ELLY				
(Strongly agree/agree) activities component helped with …	weight goal	10 (29)	10 (25)	20 (27) (17, 38)
	well-being goal	17 (49)	18 (45)	35 (47) (36, 59)
	personal goal	20 (57)	19 (48)	39 (52) (40, 64)
Loyalty card n (%)				
Participants who engaged with the loyalty card scheme achieving at least 9/12 weeks of stamps		25 (71)	30 (75)	55 (73) (62, 83)
(Strongly agree/agree) the reward was an appropriate amount		24 (69)	28 (70)	52 (69) (58, 79)
(Strongly agree/agree) the timing of the reward was appropriate (end of 12 weeks)		23 (66)	30 (60)	53 (71) (59, 81)
(Strongly agree/agree) I made new friends as a result of the loyalty card		20 (57)	25 (63)	45 (60) (48, 71)
(Strongly agree/agree) the loyalty card helped me feel more part of my community		20 (57)	21 (53)	41 (55) (43, 66)
(Strongly agree/agree) the loyalty card kept me motivated		20 (57)	24 (60)	44 (59) (47, 70)
(Strongly agree/agree) the loyalty card was an important part of ELLY		22 (63)	27 (68)	49 (65) (53, 76)
(Strongly agree/agree) loyalty card component helped with…	weight goal	11 (31)	12 (30)	23 (31) (21, 42)
	well-being goal	19 (54)	21 (53)	40 (53) (41, 65)
	personal goal	21 (60)	22 (55)	43 (57) (45, 69)
Goal setting n (%)				
Weight goal set		28 (80)	36 (90)	64 (85) (75, 93)
Well-being goal set		30 (86)	36 (90)	66 (88) (78, 94)
Personal goal set		29 (83)	36 (90)	65 (87) (77, 93)
Information resources, self-monitoring of weight and well-being n (%)				
Engagement with self-reporting of weight via ELLY website				0 (0)

#### Soup provision

Despite good engagement in ELLY soup (49/75 (65%)), participant questionnaires provided no consensus on its popularity. A significant proportion of participants indicated that they strongly agreed/agreed that ‘getting the soup was very convenient’ (33 (44%)), that ‘I made new friends as a result of ELLY soup’ (36 (48%)) and that the soup component ‘helped me feel more part of my community’ (35 (46%)). These findings were consistent across both communities. Overall, £877.44 was spent on soup ingredients for 73 participants over 12 weeks (mean average cost of soup ingredients over the 12 weeks: £12.02 per participant).

Participant interviews showed disparate views on ELLY soup; however, the majority reported they found the soup element to be positive and/or beneficial to themselves and others, including being easy and convenient to access. While it was felt that ELLY soup might support participants with dietary goals and an opportunity for healthy eating, only one participant (from C1) reported the soup helped them achieve their goal of weight loss and healthy eating. Food insecurity was an important element that ELLY soup addressed:

I also got to eat something rather than just skipping meals. This is another thing, because I skip meals and things like that [C2, P34]

ELLY soup was also recognised as an opportunity for social interaction and connection with others:

…the soup helped me because I was coming in here to pick it up and it was a direct link with people because the Covid [pandemic]…it was a long time…it made me, I won’t say nervous but unsure of mixing with people again [C2, P19]

Interviewees from both communities reported similar barriers to accessing the soup, most notably not liking the soup or there being a lack of variety of other foods available. Interviewees from C1 stated that they did not like the soup element due to the table setup at the venue, which limited opportunities for socialising with others and meeting new people. One interviewee from C2 felt the soup option was not inclusive of other cultures from across the community. Others reported that they could not attend the soup due to the time and dates it operated.

#### Community assets

Community assets signposted to by ELLY, where participants could get their ELLY loyalty card stamped, were well engaged with (55, 73%). Participants reported they strongly agreed/agreed that ‘I made new friends as a result of the activities’ (46, 61%), the activities ‘helped me feel more part of the community’ (49, 65.3%) and ‘the activities kept me motivated’ (51, 68%). Participants also strongly agreed/agreed that the activities component helped with their personal goal (39, 52%) but less so supporting weight (20, 26%) and well-being (35, 46%) goals. A majority of individuals in community C1 and just under half of individuals in C2 strongly agreed/agreed that they had engaged in more assets than they had before ELLY (C1: 24/35, 69%; C2: 18/40, 45%) and had tried new assets during ELLY (C1: 23/35, 66%; C2: 19/40, 48%).

Interview data showed that across both communities, the range of assets available was overall found to be good, well-advertised and easily accessible. A key facilitator was found to be the welcoming nature of staff and volunteers at assets:

I think just people were very welcoming, which was amazing, I think in all of the groups that I attended they were very, very welcoming [C1, P19]

Further, participants reported that having assets that were free to attend and walking distance from home were beneficial for accessibility, especially for those with little money.

[asset name] was just literally at the bottom of my street…that was the easiest because it wasn’t too far to walk [C1, P30]

Barriers reported by interviewees across both communities were related to individuals’ inability to attend assets due to employment or caring commitments and times not fitting well with their daily schedules.

…having some activities on a Monday, my day off, would have helped…the evenings are consumed by kids’ clubs… so I wouldn’t have managed [C1, P24]

A further barrier experienced in both communities was a lack of assets for different interests, ages or genders.

A lot of them were for older people, I would go to some of the clubs, I looked at them and I would go in and would be like, yes, no and I would just go. I think, guy-centred activities would have been good because most of the clubs that are run are usually female-orientated [C2, P41]

A related point made by a small number of participants was that spaces could be more inclusive to different demographic groups and needs:

…it would have been good to have spaces for people who are just in those awkward places where they don’t really fit into neat boxes…I feel like possibly those are the people who don’t fit anywhere that actually probably need it the most in some ways [C1, P19]

#### Loyalty card

Participants in both communities strongly agreed/agreed that the cash value of the loyalty card and ability to redeem it after 12 weeks of the ELLY intervention was appropriate (52 (69%) and 53 (71%), respectively). The majority of participants in both communities strongly agreed/agreed that ‘I made new friends as a result of the loyalty card’ (45, 60%), the loyalty card ‘helped me feel more part of my community’ (41, 55%) and ‘the loyalty card kept me motivated’ (44, 59%). Participants also strongly agreed/agreed that the loyalty card supported well-being (40, 53%) and personal (43, 57%) goals and was regarded as an important component of the ELLY intervention (49, 65%).

Across both communities, most interviewees found the loyalty card to be positive and beneficial to achieving their goals. Many found the loyalty card acted as an incentive to take part in more assets and was satisfying and rewarding.

It’s nice for you to look at it and go oh I’ve not been anywhere this week, I need to go and get my stamp…It was a push to go out and go somewhere because I wanted to get all the stamps [C1, P26]

11 of the interviewees stated that the loyalty card was easy to use and that the incentive offered was a good amount. Two participants from C2 stated that it was particularly beneficial for those in need.

It might be just a card and a voucher for me, but it might be something wow factor for somebody else because people do struggle, and not everybody tells you their problems [C2, P105]

Seven of the interviewees stated that the loyalty card made no difference to their attendance. Negative responses were mainly regarding practical aspects, such as the risk of losing the card or forgetting to bring it along to activities. Two respondents from C1 stated that they did not like the concept due to it being an ‘artificial encouragement’ [C1, P43, P2], encouraging people to attend for the wrong reasons, and one respondent stated that the stamp system had the potential to ‘embarrass’ people [C1, P2].

Overall, £1375 of gift card payments was made to the 55 participants (73%) who successfully acquired at least nine stamps on their loyalty card.

#### Goal setting

Across both communities, goal setting was engaged in by the majority of participants (weight goal: 64, 85%; well-being goal: 66, 88%; personal goal 65, 87%). The most popular personal goals were around meeting new people (20, 27%), setting a target weight-loss (19, 25%), doing more activities (8, 11%) and being more community focused (8, 11%).

Analysis of goals set and engagement in other ELLY components was conducted to determine if setting particular goals led to a greater likelihood of engagement in different components. For example, did participants who set a weight goal engage more with the soup cafés than those who did not? Findings suggest there was no significant difference in engagement of different ELLY components between goal setters and non-goal setters. It should be noted that the number of participants choosing not to set particular goals was low, so this finding is based on small numbers.

Interview participants from both communities reported that goal setting was a positive and helpful element of ELLY. Participants found setting goals easy and that goal setting had been useful for keeping focus and motivation.

…to know in your head that you’ve got a goal of trying to be a bit more active and lose a bit more weight and that you’ve got a timeframe for it, I think that’s a really positive thing [C1, P26]

It’s good having a focus…it kind of plants a seed and it lets me know what I need to do to get to my end goal of trying to lose some weight [C2, P34]

Eight participants specifically stated that the goal setting had helped them achieve their goals.

I feel as though they’ve [the goals] helped dramatically. So due to this, health is a lot better mentally and psychologically I’m a lot better [C1, P2]

Four C1 participants stated that they could not remember setting goals due to other things going on in their lives. One participant from C2 found the goal setting system challenging to complete due to being too busy and having personal issues.

#### Information resources and self-monitoring of weight and well-being

Interview data showed that participants liked the A4 printed ‘What’s on’ card provided in the participant packs at the start of the ELLY intervention. Three interviewees from C1 reported the programme of assets and contact details to be easily accessible online, especially through social media.

What was interesting about the ELLY project is that it was advertised in one space, whereas normally you have to rush around and try to find things in different places, so I wouldn’t necessarily have known about the [activities] [C1, P19]

Nonetheless, three participants reported that the assets themselves did not provide up to date information and/or were difficult to contact. When asked if they had looked at either the ELLY website or ELLY social media for this information, they had not.

Nobody showed up [to the activity] the times I was here. Nobody tried to contact me to find out what was wrong or anything like that [C2, P19]

The self-reporting of weight and well-being feature via the website was not used, and when questioned on this element, interviewees stated they had not felt the need to access the website.

### Feasibility and fidelity of delivering intervention components

The ELLY intervention was feasibly delivered in both community settings. Community CARP members and community champions actively supported academic researchers with recruitment activities and advertising the ELLY project. The ELLY soup component was made and delivered by community voluntary organisations in each community. In addition, in community C2, local college students supported the soup café by welcoming participants and working in the café. Assets were delivered by independent volunteers and organisations already providing activities/clubs/groups in the two communities. Data collection and analysis, provision of ELLY, the website, social media and project monitoring were undertaken by the ELLY research team. All participants who secured financial incentives received their chosen shopping card within 1 week of completing outcome measures (as stipulated). Issues that were reported were as follows: one participant reported confusion around loyalty card stamping, two participants not being able to contact activity organisers and seven not feeling welcome at some assets attended.

### Harms and unintended consequences

No harms or unintended consequences were reported.

### Effects on weight-related and well-being outcomes at 12 weeks

The effects on outcomes collected are shown in table 4.

**Table 4:** Mean (SD) change in measures collected from baseline to 12 weeks

	Mean	SD	95% CI
Weight change (kg), mean (SD)	−0.43	3.33	−1.26, 0.40
Weight change (%), mean (SD)	−0.35	3.68	−1.26, 0.56
Body mass index (kg/m^2^)	−0.15	1.26	−0.44, 0.14
EQ-5D-5L index score	0.02	0.20	−0.26, 0.07
WEMWBS	0.80	9.74	−1.44, 3.04
Social connectedness scale	0.80	14.6	−2.56, 4.16

WEMWBSWarwick-Edinburgh Mental Wellbeing Scales

### Progression to full trial

An independent study steering committee agreed that the ELLY study had demonstrated acceptability and feasibility and that the overall prespecified progression criteria were sufficiently met to support a larger-scale evaluation of the effectiveness and cost-effectiveness of ELLY.

## Discussion

### Principal findings

The ELLY study was popular, engaged citizen partners and successfully recruited 75 participants across two disadvantaged communities with an 87% (65/75) retention rate at 12-week follow-up. Community citizens living in disadvantaged areas (SIMD 1–3) formed 87% of the sample illustrating some promise for ELLY to impact on health inequalities in future. All ELLY components were acceptable to participants. The majority of ELLY components were engaged with, with the exception of the self-monitoring of weight website component, which was not used. Changes in measurable weight outcomes (decrease) and well-being outcomes (increase) were observed.

### Strengths and weaknesses

The ELLY study was effective in producing a co-designed intervention with two disadvantaged communities for use in disadvantaged communities. The intervention is underpinned by systematic review findings and theory informed and extends the evidence for use of financial incentive interventions for supporting healthy weight and well-being in disadvantaged communities.^49^ The progression criteria set by an independent study steering committee were sufficiently met to proceed to a full trial.

The feasibility study was not powered to detect effects on weight and well-being-related outcomes; therefore, findings should be interpreted with caution. Possible expectation effects, the short study time frame and assumptions of directionality of relationships were present in this research and should be addressed in its extensions. Figures provided relating to attendance at weekly soups and questionnaire data are reliant on participant self-reporting. The mean average soup cost per person of £12.02 over the 12 weeks is calculated from the cost of soup ingredients and does not account for wider opportunity costs (eg, time taken to prepare soup, electricity costs, cost of volunteering). Although communities were chosen for their disparate nature, further consideration should be given to the mix and diversity of communities in a future evaluation to maximise generalisability of findings and contribution to theory and intervention development. Careful consideration of what costs should be included in cost-effectiveness calculations, aligned to the perspective taken (eg, consideration of societal costs, public-sector costs), should be given for future evaluation of the intervention.

### Relation to other studies

ELLY findings are aligned to those reported in the review, where all studies showed community incentive interventions resulted in small improvements in BMI and/or weight or no effect. The systematic review and network meta-analysis conducted by Boonmanunt *et al*^50^ examined behavioural-economic incentive programmes for achieving goals, and reviewed the effectiveness of different strategies on incentivisation for healthy diet, weight control and physical activity. This work is important in recognising the role of self-determination theory acknowledging the impact of different social contexts and individuals’ differences on different types of motivation.

The ELLY intervention promoted autonomy and intrinsic (goal setting) and incorporated extrinsic motivation (incentivisation), social and physical opportunities, and capability to support positive health and well-being behaviour change. The ELLY intervention supports the findings of Boonmanunt *et al*^50^ that recognise the importance of social support, adding objects to/restructuring the community environment and incorporating financial rewards to support sustained behaviour change. The extensive community engagement undertaken during the ELLY project mirrors that of VanWormer *et al*^51^ where promotional strategies to recruit to the study were invested in heavily. VanWormer *et al*^51^ acknowledge that the considerable resources required may be a barrier to others wanting/being able to invest in such community engagement strategies. An emphasis on holistic health and well-being was preferred by citizens to a weight focus, reflected by the community assets offered. This finding reflects that of Glover *et al*^19^ where having to be weighed proved a barrier both to recruitment and retention for some participants. In the ELLY study, 85% selected a weight goal yet few locally provided assets focused on the required food and behavioural changes required for weight loss.

Investing in upstream public health incentive initiatives that are feasible and acceptable to communities warrants further investigation to explore their potential to reduce pressure on existing health services, including gate-keeper roles. Incorporating incentives into social prescribing may be a promising approach for highlighting and encouraging engagement with supportive community assets. A holistic approach to health well-being, rather than a focus on individual, potentially stigmatising aspects like weight or behaviour was shown in this study to be preferred by communities.

## Conclusion

This study demonstrates the feasibility of co-designing and implementing a novel community-based, incentive intervention to support healthy weight and well-being. A larger study is warranted to determine effectiveness and cost-effectiveness, with consideration of scalability. The design of a full-scale evaluation requires careful consideration to ensure its appropriateness in addressing study objectives. Community-based intervention studies can produce methodological challenges: how best to cluster across communities, how to ensure contextual differences are accounted for and how to ensure a one-size-fits-all intervention is flexible enough to address local needs, while maintaining fidelity. In the ELLY study, outcome measures prioritised by communities were multiple and of equal importance, necessitating discussion around use of coprimary outcomes in a future study. In all decisions around study design of a full-scale evaluation, ensuring equitable engagement of community citizens will be crucial in maximising study success.

## supplementary material

10.1136/bmjopen-2024-092908 online supplemental file 1

10.1136/bmjopen-2024-092908 online supplemental file 2

10.1136/bmjopen-2024-092908 online supplemental file 3

10.1136/bmjopen-2024-092908 online supplemental file 4

10.1136/bmjopen-2024-092908 online supplemental file 5

## Data Availability

Data are available upon reasonable request.
