# Supplementary figures and images for: Feasibility study of a co-designed, evidence-informed and community-based incentive intervention to promote healthy weight and well-being in disadvantaged communities in Scotland
Source: BMJ Open. 2025 Feb 20;15(2):e092908. doi: 10.1136/bmjopen-2024-092908 (PMC11843023; doi:10.1136/bmjopen-2024-092908)

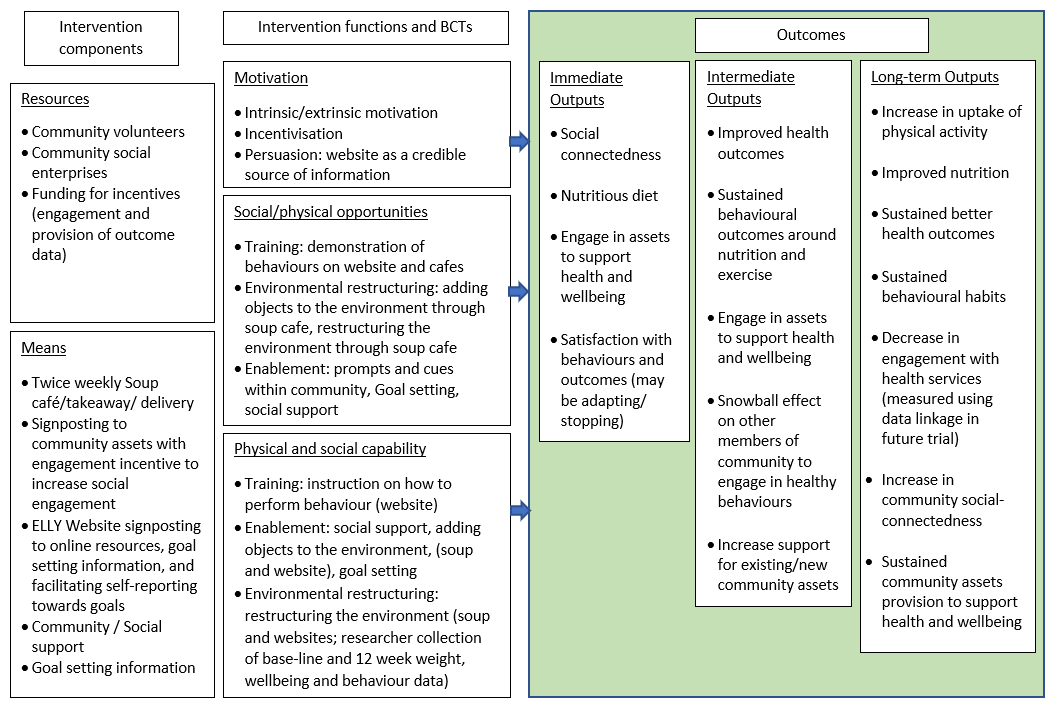
 **ELLY theory of change model**

Supplement: online supplemental file 2 [file bmjopen-15-2-s002.docx]
