# Supplementary material for: Feasibility study of a co-designed, evidence-informed and community-based incentive intervention to promote healthy weight and well-being in disadvantaged communities in Scotland
Source: BMJ Open. 2025 Feb 20;15(2):e092908. doi: 10.1136/bmjopen-2024-092908 (PMC11843023; doi:10.1136/bmjopen-2024-092908)
Supplement: online supplemental file 3 [file bmjopen-15-2-s003.docx]

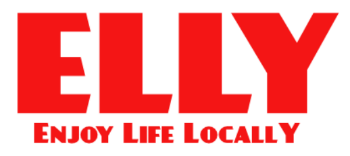


Men’s Shed
(10am-3pm)
Unit 19 F, Winchester Avenue
FREE

Mon

Tue

Wed

Thu

Fri

Sat

Sun

The Toddler Club
(9:30am-11am)
Baptist Church
FREE

Morning

Afternoon

Evening

The Hope Hub drop in
(10am-12pm)
The Hope Hub
FREE

Forget me not cafe
(10:30am-12pm & 1pm-3pm)
 Library
FREE

Knit and Knatter
(2pm-3pm)
Library
FREE

The Hope Hub drop in
(10am-12pm)
The Hope Hub
FREE

The Hope Hub drop in
(10am-12pm)
The Hope Hub
FREE

Wellbeing Wednesdays
(10:30am-11:15am)
YMCA
FREE

Young Adult Reading Group (Monthly)
(6:30pm-7:30pm)
Library
FREE

Braveheart Walk
(2pm-3pm)
Meet in Sports Centre car park
FREE

Bookbug
(11am)
Library
FREE

The Hope Hub drop in
(10am-12pm)
The Hope Hub
FREE

Words for Wellbeing (Every other week)
(11am-12pm
YMCA
FREE

Men’s Shed
(10am-3pm)
Unit 19 F, Winchester Avenue
FREE

Snowdrop Cafe
(1pm-3pm)
Westpark Church Hall
FREE

Men’s Shed
(10am-3pm)
Unit 19 F, Winchester Avenue
FREE

Men’s Shed
(10am-3pm)
Unit 19 F, Winchester Avenue
FREE

The Lymph Notes Choir
(5pm-7pm)
 Baptist Church
FREE

Feeding Families Thursdays
(4:30pm-6pm)
 Baptist Church
FREE

The Hope Hub drop in
(7pm-9pm)
The Hope Hub
FREE

Skating session
(7pm-8:30pm)
Sports Hall, C1 Centre
£5

Memory Group (Monthly)
(1:30-2:30pm)
Library
FREE

**What’s on in *Community 1*?**

 
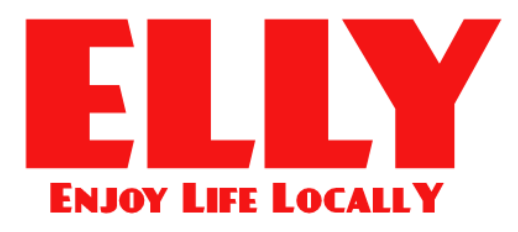


| **Activity** | **Description** |
| --- | --- |
| The Toddler Club | The Toddler Club is a parent and toddler group run by the church for little ones aged 0-3 to come along. Book your place at the link below. <https://www.dennybaptistchurch.com/events-1/the-toddler-club-2> |
| Feeding Families Thursdays | Launched in February, Feeding Families Thursdays supports local families with kids to provide a warm space, food and fun. |
| The Hope Hub drop in | The Hope Hub drop in is a great place to go along for a cuppa and a chat. Everyone is welcome. |
| Forget me not café | A friendly group that welcomes everyone, including people living with dementia. Come along for a chat, cake and to do something fun. |
| Knit and Knatter | Come along and meet other knitters, have a look through the library's knitting books, chat and swap ideas and techniques. All welcome! Bring whatever you're working on at the moment. |
| Wellbeing Wednesdays | Join others to take part in some light exercise in a relaxed and supportive environment. (Not running during October.) |
| Braveheart Walk | Do you want to become more active? Do you want to make new friendships? Do you enjoy being outdoors? Not sure of walking alone? Join us on a walk in the heart of nature with Braveheart’s free health walks designed to support adults, of all abilities, to become more physically and socially active within the community. |
| Memory Group (Monthly) | Meet other locals at the library monthly to relive old memories through photographs and stories. Contact the library for dates – *[tel no]* |
| Words for wellbeing (on every other week – 21^st^ Sep / 5^th^ Oct / 19^th^ Oct / 2^nd^ Nov / 16^th^ Nov) | The groups differ from traditional book groups in that no homework is required - just come along on the day. You'll hear short pieces of fiction, non-fiction, poetry or song lyrics and have the chance to discuss them with other participants. |
| Young Adult Reading Group (Monthly – 28^th^ Sep / 26^th^ Oct / 30^th^ Nov) | A free book club, just for young adults. It's your chance to chat about the books you love (or love to hate!). Who knows, you might meet some interesting new books and some interesting new people! |
| Bookbug | Bookbug Sessions are free, fun and friendly events for babies, toddlers and their families to enjoy together. Our sessions are suitable for ages 0+ and can be booked on Eventbrite. Book at the link below. <https://www.eventbrite.co.uk/cc/events-at-denny-library-108559> |
| Skating session | Want to get fit but find that the gym's boring and jogging's no fun? Then join us for some exercise in disguise at our adult roller-skating sessions. |
| Lymph Notes Choir | Even if you think you can't sing, enjoy the many proven benefits of singing in the social setting of a choir. Bring a friend, join us and have some fun! |
| Men’s Shed | The Men’s Shed movement started 16 years ago, as a method of counteracting the effects of boredom and isolation when faced with retirement, illness or unemployment.  Our shed workshop has a comprehensive range of tools at the disposal of members as well as social space for the essential cuppa and cake! |
| Snowdrop Cafe | What was originally a befriending idea to combat loneliness, The Snowdrop Café is open to all - a safe and happy community meeting place. Grab a coffee, a slice of cake and have a blether. Snowdrop Cafés are run by your local community, for your local community. All money donated is put back into the café running costs. |

**What’s on in *Community 2***

Evening


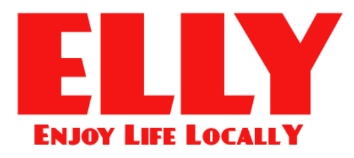


Make and Mend
(7pm-9pm)
5 Manse Place
FREE

Make and Mend
(12:30pm-2:30pm)
5 Manse Place
FREE

Sun

Sat

Fri

Thu

Wed

Tue

Mon

Step Forth Walks
(7pm-8pm)
Falkirk Stadium
FREE

Creative Writing Group
(12pm-2pm)
5 Manse Place
FREE

Wee Ones
(9:30am-11am)
WPCC
Donation of choice

Braveheart Walk
(2pm-3pm)
Callendar House
FREE

Share a craft
(10am-12pm)
WPCC
£2

Writing Group
(2pm-4pm)
WPCC
FREE

Board Games
(10am-11:30am)
5 Manse Place
FREE

Step Forth Walks
(10am-11am)
Football Stadium
FREE

Mindful Making Craft Group
(10:30am-12:30pm)
5 Manse Place
Free

Little Conversations over 50s group
(11am-12pm)
Pots Cafe
FREE

Braveheart Walk
(7pm-8pm)
Falkirk Stadium
FREE

Taekwon-Do
(6:45pm-7:45pm)
WPCC
First session FREE

Korean Kickboxing
(7:45pm-9:15pm)
WPCC
First session FREE

Move it or lose it
(11am-12pm)
WPCC
£6

Taekwon-Do
(6:45pm-7:45pm)
WPCC
First session FREE

Walk for Wellbeing
(7pm-8:30pm)
WPCC
FREE

Rainbow Muslim Women’s Group
(12:30pm-3pm)
WPCC
FREE

Share a craft
(10am-12pm)
WPCC
£2

Braveheart Walk
(1:30pm-2:30pm)
Meet in Falkirk Stadium Car Park
FREE

Korean Kickboxing
(7:30pm-8:30pm)
WPCC
First session FREE

Braveheart Walk (10:30am-11:30am)
Callendar house
FREE

Falkirk Park Run (9:30am start)
Callendar house
FREE

Morning

Afternoon

 
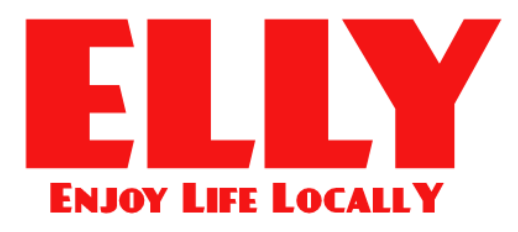


| **Activity** | **Description** |
| --- | --- |
| Walk for Wellbeing | A friendly walking group for anyone who’s mental wellbeing needs a boost. |
| Braveheart Walk | Do you want to become more active? Do you want to make new friendships? Do you enjoy being outdoors? Not sure of walking alone?  Join us on a walk in the heart of nature with Braveheart’s free health walks designed to support adults, of all abilities, to become more physically and socially active within the community.  Our friendly and welcoming walks promote social inclusion within the community, encourage the use of green space, and raise awareness of the benefits of active travel within your local area. (Thursday walk only on April-October). |
| Share a Craft | Bring a crafting activity or come along to get some ideas and see what other people are working on! If you would like to join this welcoming, lovely group with your own craft it's 10am-12pm at Community Centre. |
| Rainbow Muslim Women’s Group | Rainbow Muslim women group is a charity organisation aiming to provide social and educational opportunities to the vulnerable sector of the community, across Forth Valley Area since 1999. |
| Move it or lose it | Come and join in with others as we do some fun light exercise in a supportive environment (60+). |
| Wee Ones | Come and meet other families here at the centre. Each week will be different activities for the kids, while the parents enjoy a free cuppa! (up to 5 years old) |
| Step Forth Walks | Step Forth is our award-winning volunteer led free walking programme designed to improve your physical activity levels through walking. |
| Taekwon-Do | TaeKwon-Do is a Korean Martial Art that dates back 2000 years. TaeKwon-Do means “The art of hand and foot fighting” and is used primarily for self-defence. First session free then £30 per month (for 2 sessions a week). |
| Korean Kickboxing | Our Korean kickboxing originated from Taekwon-Do mixed with boxing. It is a self-defence and fitness contact sport that utilises kicks and punches. First session free then £30 per month (for 2 sessions a week). |
| Falkirk Park Run | A free, fun, and friendly weekly 5k community event. Walk, jog, run, volunteer or spectate – it's up to you! (You won’t get a stamp at this but if you follow the instructions on the website and sign up we will be able to see when you have run). |
| Central Wellbeing activities | Central Wellbeing run a range of activities (e.g. Make and Mend and Little Conversations over 50’s group) in the Falkirk area, many based out of their office at 5 Manse Place. Find out more about their activities ([Central Wellbeing](https://www.centralwellbeing.org/)). |
