# Supplementary material for: Feasibility study of a co-designed, evidence-informed and community-based incentive intervention to promote healthy weight and well-being in disadvantaged communities in Scotland
Source: BMJ Open. 2025 Feb 20;15(2):e092908. doi: 10.1136/bmjopen-2024-092908 (PMC11843023; doi:10.1136/bmjopen-2024-092908)
Supplement: online supplemental file 4 [file bmjopen-15-2-s004.docx]

**ELLY measurements and engagement questionnaire**

**
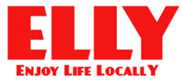
**

12 weeks: ELLY measurements and engagement questionnaire

| **Participant ID** |  |
| --- | --- |
| **Researcher name** |  |
| **Today’s date** | _ _ / _ _ / _ _ _ _  **e.g. 05 / 01 / 2021** |

*Note for interviewer: Determine participant preference for completion:*

*(preferred) To complete questionnaire themselves (with interviewer just checking complete at end)*

*To have questions read to them and interviewer record responses*

Measurements

| **Measurements (please tick)** |  | |
| --- | --- | --- |
| **Which weight measure do you prefer?** | 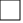 **Kg** | 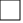 **Stones/Ibs** |

|  | **Measure 2 (12 weeks)** | **Participant**  **Initials** | **Notes** |
| --- | --- | --- | --- |
| **Weight (kg)** | **___ ___ ___ . ___ ___ kg**  **___ ___ st ___ . ___ ___ lbs** |  |  |
| *Height as recorded at baseline (cm)*  *(transfer over)* | **___ ___ ___ . ___** |  |  |
| **BMI*(Kg/m^2^)** | **___ ___ . ___ ___ kg/m^2^** |  |  |

# Engagement in local activities

Please indicate (with a tick) how often you attend the following types of activities in the last 12 weeks?

|  | 0-1 over 12 weeks | 2-4 over 12 weeks | 3-5 over 12 weeks | 6+ over 12 weeks |
| --- | --- | --- | --- | --- |
| Arts & crafts activity |  |  |  |  |
| Physical Activity group |  |  |  |  |
| Nutrition related group |  |  |  |  |
| Social related group |  |  |  |  |
| Other (please specify) |  |  |  |  |

| **Please tick the box that best describes your experience of attending local activities as part of the ELLY project.** | **Strongly disagree** | **Disagree** | **Neither agree or disagree** | **Agree** | **Strongly agree** | **Not relevant** |
| --- | --- | --- | --- | --- | --- | --- |
| I attended more activities during the project than I did before the project |  |  |  |  |  |  |
| I attended new activities during the project |  |  |  |  |  |  |
| In the last 12-weeks I attended new local activities in addition to the ones on the “what’s on” sheet |  |  |  |  |  |  |
| I feel more interested in trying out new activities as a result of the ELLY Project. |  |  |  |  |  |  |
| The activities helped me achieve the PERSONAL goal I set at the start of the ELLY project |  |  |  |  |  |  |
| The activities helped me achieve the WEIGHT goal I set at the start of the ELLY project |  |  |  |  |  |  |
| The activities helped me achieve the WELLBEING goal I set at the start of the ELLY project |  |  |  |  |  |  |
| I made new friends as a result of the activities |  |  |  |  |  |  |
| The activities helped me feel more part of my community |  |  |  |  |  |  |
| I feel like the activities kept me motivated |  |  |  |  |  |  |
| I feel the activities were an important part of the ELLY project |  |  |  |  |  |  |

If you disagreed with any of the statements above, we would be interested to hear why _____________________________________________________________________________________________________________________________________________________________________________________________________________________

If you answered not relevant to any of the questions above, we would be interested to hear why it was not relevant _____________________________________________________________________________________________________________________________________________________________________________________________________________________

In summary, how best would you describe your experience of taking part in the activities? _____________________________________________________________________________________________________________________________________________________________________________________________________________________

Is there anything else about the activities you would like to share with us? (e.g. if you answered strongly disagree to any of the above you might like to share alternatives or suggestions for improvements)

___________________________________________________________________________________________________________________________________________________________________________________________________________________________________________________________________________________________________________________________________________________________________

**ELLY Loyalty card and reward**

| **Please tick the box that best describes your experience of the loyalty card and reward as part of the ELLY project.** | **Strongly disagree** | **Disagree** | **Neither agree or disagree** | **Agree** | **Strongly agree** | **Not relevant** |
| --- | --- | --- | --- | --- | --- | --- |
| I think the reward was an appropriate amount |  |  |  |  |  |  |
| I think the timing of the reward was appropriate (at the end of the 12-weeks) |  |  |  |  |  |  |
| The loyalty card and reward helped me achieve the PERSONAL goal I set at the start of the ELLY project |  |  |  |  |  |  |
| The loyalty card and reward helped me achieve the WEIGHT goal I set at the start of the ELLY project |  |  |  |  |  |  |
| The loyalty card and reward helped me achieve the WELLBEING goal I set at the start of the ELLY project |  |  |  |  |  |  |
| I made new friends as a result of the loyalty card and reward |  |  |  |  |  |  |
| The loyalty card and reward made me feel more part of my community |  |  |  |  |  |  |
| I feel like the loyalty card and reward kept me motivated |  |  |  |  |  |  |
| I feel the loyalty card and reward were an important part of the ELLY project |  |  |  |  |  |  |

If you disagreed with any of the statements above, we would be interested to hear why _____________________________________________________________________________________________________________________________________________________________________________________________________________________

If you answered not relevant to any of the questions above – we would be interested to hear why it was not relevant _____________________________________________________________________________________________________________________________________________________________________________________________________________________

In summary, how best would you describe your experience of the loyalty card and reward? _____________________________________________________________________________________________________________________________________________________________________________________________________________________

Is there anything else about the loyalty card and reward you would like to share with us? (e.g. if you answered strongly disagree to any of the above you might like to share alternatives or suggestions for improvements)

___________________________________________________________________________________________________________________________________________________________________________________________________________________________________________________________________________________________________________________________________________________________________

**ELLY SOUP**

If you took up the offer of soup twice a week, how did you get your soup? (please tick all that apply)

| Sit in at café, twice weekly |  |
| --- | --- |
| Collect soup twice weekly from cafe |  |
| Collect 2 portions of soup once a week from cafe |  |
| Delivered to house |  |
| Other (please state) |  |

| **Please tick the box that best describes your experience of the twice weekly free soup you received as part of the ELLY project.** | **Strongly disagree** | **Disagree** | **Neither agree or disagree** | **Agree** | **Strongly agree** | **Not relevant** |
| --- | --- | --- | --- | --- | --- | --- |
| Getting soup twice a week was very convenient |  |  |  |  |  |  |
| The twice weekly soup helped me achieve the PERSONAL goal I set at the start of the ELLY project |  |  |  |  |  |  |
| The twice weekly soup helped me achieve the WEIGHT goal I set at the start of the ELLY project |  |  |  |  |  |  |
| The twice weekly soup helped me achieve the WELLBEING goal I set at the start of the ELLY project |  |  |  |  |  |  |
| I made new friends as a result of ELLY soup |  |  |  |  |  |  |
| ELLY soup made me feel more part of my community |  |  |  |  |  |  |
| I feel like ELLY soup kept me motivated |  |  |  |  |  |  |
| I feel ELLY soup was an important part of the ELLY project |  |  |  |  |  |  |
| I made new friends as a result of the twice weekly soup |  |  |  |  |  |  |
| The twice weekly soup helped me feel more part of my community |  |  |  |  |  |  |
| I feel the twice weekly soup was an important part of the ELLY project |  |  |  |  |  |  |

If you disagreed with any of the statements above, we would be interested to hear why _____________________________________________________________________________________________________________________________________________________________________________________________________________________

If you answered not relevant to any of the questions above – we would be interested to hear why it was not relevant _____________________________________________________________________________________________________________________________________________________________________________________________________________________

In summary, how best would you describe your experience of the ELLY soup twice weekly _____________________________________________________________________________________________________________________________________________________________________________________________________________________

Is there anything else about the ELLY twice weekly soup you would like to share with us? (e.g. if you answered strongly disagree to any of the above you might like to share alternatives or suggestions for improvements) ___________________________________________________________________________________________________________________________________________________________________________________________________________________________________________________________________________________________________________________________________________________________________

**ELLY project overall**

What aspects of the project do think were particularly successful? _____________________________________________________________________________________________________________________________________________________________________________________________________________________

_______________________________________________________________________

What aspects of the project were challenging or unsuccessful? _____________________________________________________________________________________________________________________________________________________________________________________________________________________

_______________________________________________________________________

What would you suggest could improve the project for future participants? _____________________________________________________________________________________________________________________________________________________________________________________________________________________

_______________________________________________________________________

Thinking about the goals you set, what are your thoughts on where you are with these now? _____________________________________________________________________________________________________________________________________________________________________________________________________________________

_______________________________________________________________________

In summary, how best would you describe your experience of taking part in ELLY? _____________________________________________________________________________________________________________________________________________________________________________________________________________________

_______________________________________________________________________

Is there anything else about ELLY you would like to share with us?

___________________________________________________________________________________________________________________________________________________________________________________________________________________________________________________________________________________________________________________________________________________________________

**Thank you for your time completing this questionnaire.**

**Your feedback is really important to us and will help shape future projects.**
