# Supplementary material for: Feasibility study of a co-designed, evidence-informed and community-based incentive intervention to promote healthy weight and well-being in disadvantaged communities in Scotland
Source: BMJ Open. 2025 Feb 20;15(2):e092908. doi: 10.1136/bmjopen-2024-092908 (PMC11843023; doi:10.1136/bmjopen-2024-092908)
Supplement: Supplementary file 6 [file bmjopen-2024-092908.reviewer_comments.pdf]

PEER REVIEW HISTORY

BMJ Open publishes all reviews undertaken for accepted manuscripts. Reviewers are asked to complete a checklist review form and are provided with free text boxes to elaborate on their assessment. These free text comments are reproduced below.

ARTICLE DETAILS

Title (Provisional)

Feasibility study of a co-designed, evidence-informed and community-based incentive intervention to promote healthy weight and wellbeing in disadvantaged communities in Scotland

Authors

Cowie, Julie; Findlay, Scott; Archibald, Rhonda; Currie, Sinead; Campbell, Pauline; Hutcheon, Danielle; van der Pol, Marjon; MacLennan, Graeme; Cook, Elizabeth; Lock, Bette; Hoddinott, Pat

---

VERSION 1 - REVIEW

**Reviewer**	**1**
**Name**	**Daley, Amanda**
**Affiliation**	**Loughborough University, School of Sport, Exercise and Health Sciences**
**Date**	**14-Nov-2024**
**COI**	**I understand and consent to the named publication of this review.**

General

The authors have presented an interesting and much needed study. The combination and quantitative and qualitative methods provided a nice combination of data and perspectives. Some specific comments are outlined below and the authors are encouraged to pay particular attention to detail and consistency.

Specific comments

Abstract

The abstract is very difficult to follow, and important information is missing. The intervention description is not well expressed and grammatically incorrect in places. Not clear what unconditional soup is – is this supposed to be unlimited soup? This reviewer isn't clear what is meant be unconditional here and in the main manuscript. It is stated that intervention components are an outcome measure, but this doesn't make sense. Healthy body weight is

mentioned as an outcome but this is not an outcome in itself – is this referring to the proportion with a healthy body weight or something else. What was the target for recruitment and what was the % of this that was achieved – this is not well expressed. Reference is made to body mass but this isn't an outcome? Body mass index is an outcome. The conclusion refers to the Elly trial but this is the first time the name is used in the abstract? There are words missing in the abstract that need attention and the abstract needs greater attention to detail overall. Were all participants adults? These comments would apply to the main manuscript as well.

Article summary

Not sure what is meant by 'a holistic approach – what is holistic in the intervention?

Reference is made to the intervention having a measurable impact on weight, but weight isn't one of the outcomes – according to the abstract? The outcome stated is BMI which is not the same thing.

Methods

Was the research pre-registered on a publicly available database in line with good practice?

How were the two disadvantaged communities selected and why? How do these communities compare to other disadvantaged communities? How can we be sure there is nothing 'special' about these communities? Have the research team worked with the communities before or are they completely 'fresh'. Readers need to be assured that bias is not at play here and the description of the communities is very vague. What is public housing? Is this social housing?

The stop-go criteria are inconsistent – for example Table 1 states that 'feasibility of recruiting 60 participants within 3 months but over the page it states that feasibility was judged according to recruiting at least 30 citizens in each community in 3 months – again this is not the same outcome. This is just example of inconsistently in the manuscript and the authors really do need greater attention to detail throughout. Were the criteria 'drop dead' in that it was yes or no? The feasibility outcomes are described but criteria are presented for most of the outcomes in the table on page 7. So it is difficult to know whether feasibility and acceptability was actually met.

Adverse events were recorded – what events specifically were assessed and collected?

Page 9 is a lot of dense text.

Why was 60 selected as the sample size? Why not 50 or 70 for example? The use of the term 'event rate' in this context is unusual? Is this referring to loss to follow up?

The qualitative data analysis section is quite short and some detail here would be helpful – some space from the results could be used.

Several outcomes are referred to in the manuscript, but it is not clear what the intended primary outcome would be for the phase 3 trial? Is it weight or something else?

How was retention defined? Was this based on questionnaire completion or something else?

Results

Table 2 probably doesn't need all of the height and weight outcomes, similarly for working status – some lines could be combined

A lot of data is presented, much of which could be simplified

The cost of the soup is £12.02 – needs to be made clear throughout the manuscript what this is referring to – is this per week or over 12 weeks?

Discussion

SDT is mentioned but what is the theoretical basis of the intervention?

No strengths or weakness are presented

Reviewer **2**

Name **Koutoukidis, Dimitrios**

Affiliation **University of Oxford, Nuffield Department of Primary Care Health Sciences**

Date **02-Dec-2024**

COI **I understand and consent to the named publication of this review.**

Thank you for the opportunity to review this interesting and well-written paper. It is a well conducted feasibility trial with a rounded approach to process evaluation. I think the results will add to the evidence base. A few comments below for consideration.

Abstract

Intervention: unclear what "unconditional soup" and "assets" are, suggest reword the whole section for clarity.

Conclusion not supported by results section - no data on weight or well being in the results section. I suggest this is not overstated.

"Goal setting options (personal, weight, wellbeing goals) discussed with participants at baseline;" this needs more clarity - how long was the baseline counselling, did it involve anything beyond goal setting, what does "discussed" mean (were they explicitly asked to set goals or given simply the option to)?. What goals were these? goals for behaviours, for behavioural outcomes or both? How specific was it? Was it accompanied by an action plan?

I can see how nutrition-related groups and PA are the expected choices of groups to help with healthy weight - but what about arts and crafts and social groups - how are these linked

with the intervention aims? One aspect is wellbeing but did they also contribute to healthy weight?

It would be useful to present data on Table 3 split by those with weight loss goals, personal goals, and wellbeing goals in the supplement, So that we can see if eg those setting weight loss goals found the soup provision more helpful.

Indicative effects on healthy weight and wellbeing at 12-weeks: I suggest the authors reword this section by simply providing the estimates descriptively but not claiming potential/promising effects, as the 95% CIs are crossing 0. I would replace BMI with weight change. It would be useful to specify in the stats section the method with which the CIs were calculated. It is unclear how missing data were handled for weight.

Page 22 line 32: suggest replace conditions with aspects - weight or behaviours are not conditions.

Conclusion: "The design of a full scale evaluation requires careful consideration to ensure its appropriateness in addressing study objectives":this is fair but there are no lessons learnt from the feasibility study in the discussion at the moment. It would be good to elaborate on those in the discussion.

Typos

Page 5 line 37: weight mentioned twice

Page 22 line 31: health and wellbeing

Spaces before commas in a few places

VERSION 1 - AUTHOR RESPONSE

**Reviewer 1 comments (Prof. Amanda Daley, Loughborough University)**	
Comments	Response
General<br>The authors have presented an interesting and much needed study. The combination and quantitative and qualitative methods provided a nice combination of data and perspectives. Some specific comments are outlined below and the authors are	We appreciate the reviewer's comments and recognition that our work is interesting and much needed.<br><br>Thank you for your helpful comments and suggestions. We have addressed these below.

encouraged to pay particular attention to detail and consistency.	
**Abstract**	
The abstract is very difficult to follow, and important information is missing. The intervention description is not well expressed and grammatically incorrect in places. Not clear what unconditional soup is – is this supposed to be unlimited soup? This reviewer isn't clear what is meant be unconditional here and in the main manuscript.	Section Abstract has been reworded to provide better clarity and readability.<br><br>Unconditional soup means that soup provision is provided freely, without condition or need for payment. We have reworded our description of the Soup component (Section Abstract/Intervention Components) as follows: 'The Enjoy Life LocallY (ELLY) intervention comprised free soup twice weekly (café/delivery/pickup)'.<br>In addition, we have reworded the 'Intervention Components' section of the manuscript to clarify soup is free: 'ELLY is a 12-week intervention comprising of: (i) provision of free soup twice weekly (café/delivery to home/pickup) for all participants'.<br><br>We recognise clarity around the components of the intervention is important. As such, we have created an addition figure, Figure 2, which represents the TIDieR description of the intervention to provide the reader with headline information about ELLY. The figure will be included and is referenced in Section Intervention Components.
It is stated that intervention components are an outcome measure, but this doesn't make sense. Healthy body weight is mentioned as an outcome but this is not an outcome in itself – is this referring to the proportion with a healthy body weight or something else.	Section Abstract/Outcomes has been re-written to provide greater clarity.<br><br>Throughout the manuscript, we have sought to clarify that we use 'healthy weight' (a term chosen by our communities) to represent positive weight-related outcomes (weight and BMI). In addition, we have provided more specific information regarding outcomes, splitting the Abstract/Outcomes section into primary and secondary outcomes: 'Primary outcomes - feasibility of recruitment, retention and engagement. Acceptability of intervention components, assessed by self-reported questionnaires and interviews. Secondary outcomes – feasibility of collecting outcomes prioritised by communities for a future trial: health-related quality-of-life (EQ-5D-5L), mental wellbeing (WEMWBS), connectedness (Social Connectedness Scale) and weight-related measures (weight, Body Mass Index (BMI)).'
What was the target for recruitment and what was the % of this that was achieved – this is not well expressed.	We have added in detail of target recruitment figure and the percentage achieved (Section Abstract/Results): 'Over 3 months, 75 community citizens (35 citizens in C1, 40 citizens in C2) were recruited (125% of target recruitment of 60 participants (117% of 30 participants C1 target, 133% of 30 participants C2 target),'
Reference is made to body mass but this isn't an outcome? Body mass index is an outcome.	Body Mass has been corrected to Body Mass Index (Section Abstract/Outcomes).

<table>
<tr><td>The conclusion refers to the Elly trial but this is the first time the name is used in the abstract?</td><td>The intervention name is now introduced in the PICO description (Abstract/Intervention): ‘Intervention: The Enjoy Life LocallY (ELLY) intervention comprised…’</td></tr>
<tr><td>There are words missing in the abstract that need attention and the abstract needs greater attention to detail overall.</td><td>The abstract has been rewritten to provide greater detail and clarity about the work undertaken.</td></tr>
<tr><td>Were all participants adults? These comments would apply to the main manuscript as well.</td><td>Participants were adults. This detail has been added to the following sections:
Abstract/Participants: ‘Participants: Eligible adult (18 years or over) community members recruited through community outreach.’
Methods/Eligibility criteria: ‘Inclusion criteria: Any adult (aged 18 or over)’</td></tr>
<tr><td>Article summary</td><td></td></tr>
<tr><td>Not sure what is meant by ‘a holistic approach – what is holistic in the intervention?</td><td>This bullet point has been removed following guidance from the editor about appropriate bullets for this Section of the paper.
We have added clarity about what we mean by a ‘holistic’ approach where the term is first introduced (Section Methods/Intervention Components, paragraph 2):
‘Community consultation indicated that an intervention focusing solely on weight was felt stigmatising and not inclusive of all community citizens. Citizens expressed a desire for an intervention to support them as a “whole person” (recognising mental, physical, social, spiritual aspects), rather than a focus on one component alone. The resulting intervention adopts a holistic approach to supporting healthy weight and wellbeing, acting as a connector to existing assets and promoting autonomy.’</td></tr>
<tr><td>Reference is made to the intervention having a measurable impact on weight, but weight isn’t one of the outcomes – according to the abstract? The outcome stated is BMI which is not the same thing.</td><td>We have separated out weight-related outcomes (weight and BMI) to provide greater clarity about what was measured. The manuscript has been updated throughout to refer specifically to both weight-related outcomes. Data relating to weight change (actual and %) in addition to BMI is now provided in Table 4 (shown below) (Section Effects on weight-related and wellbeing outcomes at 12-weeks).

<table>
<tr><th></th><th>Mean</th><th>SD</th><th>95% CI</th></tr>
<tr><td>Weight change (kg), mean (SD)</td><td>-0.43</td><td>3.33</td><td>-1.26, 0.40</td></tr>
<tr><td>Weight change (%), mean (SD)</td><td>-0.35</td><td>3.68</td><td>-1.26, 0.56</td></tr>
<tr><td>Body Mass Index (kg/m^2)</td><td>-0.15</td><td>1.26</td><td>-0.44, 0.14</td></tr>
<tr><td>EQ-5D-5L index score</td><td>0.02</td><td>0.20</td><td>-0.26, 0.07</td></tr>
<tr><td>WEMWBS</td><td>0.80</td><td>9.74</td><td>-1.44, 3.04</td></tr>
<tr><td>Social connectedness scale</td><td>0.80</td><td>14.6</td><td>-2.56, 4.16</td></tr>
</table></td></tr>
<tr><td>Methods</td><td></td></tr>
<tr><td>1) Was the research pre-registered on a</td><td>The systematic review conducted as part of the research was registered on PROSPERO (CRD42022343239), however the main study was not pre-</td></tr>
</table>

publicly available database in line with good practice?	registered. We recognise this was an oversight on our part when the project began in 2022.
How were the two disadvantaged communities selected and why? How do these communities compare to other disadvantaged communities? How can we be sure there is nothing 'special' about these communities? Have the research team worked with the communities before or are they completely 'fresh'. Readers need to be assured that bias is not at play here and the description of the communities is very vague.	Further detail about the communities involved, how they were chosen and researchers' relationships with the communities has been added to the following sections:<br><br>Abstract/Setting: 'Setting: Two communities in Scotland experiencing high levels of disadvantage according to the Scottish Index for Multiple Deprivation (SIMD). Community C1 is in a large rural area with small town centre (population ~1.5K), community C2 is a small and urban community (population ~9K), enabling contextual comparison.'<br><br>Methods/Setting: 'The academic team was approached by NHS Forth Valley Public Health Nutrition Team (FVPHNT) as healthy weight was a concern raised by citizens through the Local Authority Community Planning Process across disadvantaged communities in the region. Two disadvantaged communities (SIMD 1-3 (quintile) in Forth Valley were chosen that were disparate in nature but felt representative of communities across the region and more widely, across Scotland. Researchers had no engagement with either community prior to the study commencing.<br>Housing in both communities predominantly comprised of public (social) housing. Assets in both communities are local activities, groups and clubs focusing on arts and crafts, physical activity, nutrition, and socialising. Community (C1) is a small rural town, with population of approximately 8000 people. SIMD levels range from 1-3 (quintile) in the target area, with more affluent areas (SIMD 4-5) on the periphery. The community partners operated on two separate sides of the town and had no prior interactions. Local assets are based predominately at community hubs, the local library, and church. The largest supermarket is a 10-minute walk from the town centre with the alternative being local shops.<br>Community 2 (C2) is a small and urban community, with population of approximately 9000 people. SIMD levels range from 1-2 (quintile). Local assets are mainly based at the community centre operated by our community partner. A retail park (and the closest supermarket) is a 20-minute walk away with a small grocery shop and petrol station located in the target area.'
What is public housing? Is this social housing?	We were mindful to use a phrase that would be understood by international readers, and reviewing international publications, public housing was a widely used term. However, we have added a qualifier (social housing) in brackets (Section Methods/Study Design/Setting): 'Housing in both communities predominantly comprised of public (social) housing'
The stop-go criteria are inconsistent – for example Table 1 states that 'feasibility of recruiting 60	We have rectified the inconsistency and sought to clarify our target as 60 participants overall (30 in each community). Clarification has been added to the following sections:

participants within 3 months but over the page it states that feasibility was judged according to recruiting at least 30 citizens in each community in 3 months – again this is not the same outcome. This is just example of inconsistently in the manuscript and the authors really do need greater attention to detail throughout.	Abstract/Results: 'Over 3 months, 75 community citizens (35 citizens in C1, 40 citizens in C2) were recruited (125% of target recruitment of 60 participants (117% of 30 participants C1 target, 133% of 30 participants C2 target)'<br><br>Table 1: 'Feasibility of recruiting 60 participants (30 per community) within 3 months'<br><br>Methods/Sample size: 'The study aimed to recruit 60 participants (30 at each community) to be sufficient in testing feasibility…'
Were the criteria 'drop dead' in that it was yes or no? The feasibility outcomes are described but criteria are presented for most of the outcomes in the table on page 7. So it is difficult to know whether feasibility and acceptability was actually met.	The criteria were not hard and fast rules, we treated these criteria as targets. In retrospect, green, amber, and red zones, in line with internal NIHR pilot and feasibility criteria might have been more useful to convey this. We saw these targets as being aspirational, by that we mean if targets were met or exceeded, we would be confident a larger trial would look quite similar and have a high chance of being feasible. If we got close to targets but did not meet or exceed those, we would look at reasons why and judge whether learning from any emerging reasons would lead to changes that would increase the changes of a larger trial being feasible.
Adverse events were recorded – what events specifically were assessed and collected?	Adverse events related to participants becoming unwell or distressed, or disclosing information relating to a health condition during the study. This information has been added to Section Methods/Outcome assessment, paragraph 2: 'Information on adverse events was recorded at assessments or at the time of reporting if during the 12-week intervention. Adverse events related to participants becoming unwell or distressed, or disclosing information relating to a health condition during the study.'
Page 9 is a lot of dense text.	We have separated engagement with ELLY activities, engagement with ELLY soup, and acceptability of all components into separate bulleted sections to aid with clarity and readability.
Why was 60 selected as the sample size? Why not 50 or 70 for example? The use of the term 'event rate' in this context is	We agree the term 'event rate' is perhaps unusual in this context and have replaced it with 'proportion':<br>Methods/Sample size: 'The study aimed to recruit 60 participants (30 at each community) to be sufficient in testing feasibility based on an estimated proportion of 5% for unforeseen problems (assuming a 95% confidence level).[46]'

unusual? Is this referring to loss to follow up?	A sample size of 60 was chosen following the pilot sample size rule of thumb proposed in Viechtbauer et al (2015) (Doi: https://doi.org/10.1016/j.jclinepi.2015.04.014). The authors state that a sample size of 59 is sufficient to detect any unforeseen problems with 95% confidence in pilot studies (except problems with follow-up). A sample size of 60 was selected to provide a target recruitment of 30 in each community.
The qualitative data analysis section is quite short and some detail here would be helpful – some space from the results could be used.	We have addressed this comment and now include further data from our qualitative results. (Section Acceptability of intervention components)
Several outcomes are referred to in the manuscript, but it is not clear what the intended primary outcome would be for the phase 3 trial? Is it weight or something else?	Outcomes that the communities prioritised for a future trial of ELLY included wellbeing and weight related outcomes. We would therefore recommend that any future trial should measure all these essential outcomes and the primary outcome should comprise of these multiple measures. We have added reflection on future trial considerations in Section Conclusion:<br>'Community-based intervention studies can produce methodological challenges: how best to cluster across communities, how to ensure contextual differences are accounted for and how to ensuring a one-size-fits-all intervention is flexible enough to address local needs, whilst maintaining fidelity. In the ELLY study, outcome measured prioritised by communities were multiple and of equal importance, necessitating discussion around use of co-primary outcomes in a future study. In all decisions around study design of a full scale evaluation, ensuring equitable engagement of community citizens will be crucial in maximising study success.'
How was retention defined? Was this based on questionnaire completion or something else?	Retention was defined as the number of participants completing 12 week outcome measures. This detail has been added to the following sections:<br>Abstract/Results: 'Retention at 12 weeks, defined by completion of outcome measures at 12 weeks, was 65 (87%)'<br>Methods/Recruitment and retention: 'Retention at 12 weeks, defined by completion of the 12 week outcome measures assessment was completed by 65/75 participants (87%) with minimal difference in retention between communities (C1 30/35 (86%) retention, C2 35/40 (88%) retention).'
Results<br>1) Table 2 probably doesn't need all of the height and weight outcomes, similarly for working status – some lines could be combined. A lot of	1) Table 2 has now been simplified by combining some data rows. Specifically, BMI, SIMD, marital status and working status categories have combined data rows as shown below:<br><table><tr><th></th><th>C1</th><th>C2 n=40</th><th>Total</th></tr><tr><td>**Age (years), mean (SD)**</td><td>56.5</td><td>50.4</td><td>53.3 (17)</td></tr><tr><td>**Gender, n (%)**</td><td></td><td></td><td></td></tr><tr><td>Female</td><td>29 (83)</td><td>34 (85)</td><td>63 (84)</td></tr><tr><td>Male</td><td>6 (17)</td><td>6 (15)</td><td>12 (16)</td></tr></table>

data is presented, much of which could be simplified
2) The cost of the soup is £12.02 – needs to be made clear throughout the manuscript what this is referring to – is this per week or over 12 weeks?

**Height (cm), mean (SD)**	162.1	163.9	163 (8)
**Weight (kg), mean (SD)**	83.9	85.6	84.8 (20)
**BMI (kg/m2), mean (SD)**	32.1 (7)	31.7 (8)	31.9 (7)
**BMI (kg/m2), categories, n (%)**			
Healthy weight (18.5 <= Body Mass Index	5 (14)	7 (18)	12 (16)
Overweight (25.0 <= Body Mass Index <=	10 (29)	6 (15)	16 (21)
Obesity/Morbid Obesity (30.0 <= Body Mass	20 (57)	26 (65)	46 (66)
Underweight (Body Mass Index < 18.5)	0 (0)	1 (3)	1 (1)
**SIMD deprivation category, n (%)**			
SIMD 1 (most disadvantaged)	11 (31)	7 (18)	18 (24)
SIMD 2	10 (29)	20 (50)	30 (40)
SIMD 3	10 (29)	7 (18)	17 (23)
SIMD 4/5 (least disadvantaged)	4 (11)	6 (15)	10 (13)
**Marital status, n (%)**			
Married/civil partnership/cohabiting	15 (43)	17 (43)	32 (43)
Separated/Widowed/Divorced	9 (26)	13 (33)	22(29)
Single (never married and never registered	10 (29)	8 (20)	18 (24)
Prefer not to say	1 (3)	2 (5)	3 (4)
**Comorbidities, n (%)**			
A stroke (including mini-stroke)	2 (6)	3 (8)	5 (7)
High blood pressure	12 (34)	10 (25)	22 (29)
A heart condition such as angina or atrial	8 (23)	6 (15)	14 (19)
Diabetes	11 (31)	3 (8)	14 (19)
Cancer	3 (9)	4 (10)	7 (9)
Arthritis	9 (26)	12 (30)	21 (28)
A mental health condition	14 (40)	18 (45)	32 (43)
None of the above	10 (29)	14 (35)	24 (32)
Report a single comorbidity	9 (26)	12 (30)	21 (28)
Report multiple long term conditions	16 (46)	14 (35)	30 (40)
**Ethnic group, n (%)**			
Asian or Asian British	2 (6)	7 (18)	9 (12)
Black, African, Caribbean or Black British	0 (0)	1 (3)	1 (1)
Mixed or multiple ethnic groups	0 (0)	1 (3)	1 (1)
Other Ethnic Group	0 (0)	2 (5)	2 (3)
White	33 (94)	29 (73)	62 (83)
**Education, n (%)**			
At degree level or above	2 (6)	10 (25)	12 (16)
Another kind of qualification	21 (60)	23 (58)	44 (59))
Prefer not to say	2 (6)	1 (3)	3 (4)
No formal qualifications	6 (17)	3 (8)	9 (12)
Not reported	4 (11)	3 (8)	7 (10)
**Household status**			
Household size, mean (SD)	2.4 (1)	2.8 (2)	2.6 (2)
Living alone, n (%)	10 (29)	13 (33)	23 (31)
**Working status, n (%)**			
Have paid job - Full time (30+ hours per	2 (6)	4 (10)	6 (8)
Have paid job - Part time (29 hours or less)	1 (3)	7 (18)	8 (11)
Unemployed and seeking work	2 (6)	4 (10)	6 (8)
Retired	16 (46)	9 (23)	25 (33)

<table>
<tr><td></td><td>
<table>
<tr><td>Full time student</td><td>0 (0)</td><td>1 (3)</td><td>1 (1)</td></tr>
<tr><td>Not in paid work due to long term</td><td>9 (26)</td><td>11 (28)</td><td>20(27)</td></tr>
<tr><td>Not reported/Other/Prefer not to say</td><td>5 (14)</td><td>4 (10)</td><td>9 (12)</td></tr>
</table>

2) The average soup cost per person is over the 12 weeks. This has been clarified in the following sections:
Abstract/Results: ‘The mean average cost of the soup ingredients, per participant, over the 12 weeks was £12.02.’
Soup provision: ‘(mean average cost of soup ingredients over the 12 weeks: £12.02 per participant)’

Discussion/Strengths and weaknesses: ‘The mean average soup cost per person of £12.02 over the 12 weeks is calculated from the cost of soup ingredients and does not account for wider opportunity costs (e.g. time taken to prepare soup, electricity costs, cost of volunteering).’
</td></tr>
<tr><td>Discussion</td><td></td></tr>
<tr><td>SDT is mentioned but what is the theoretical basis of the intervention?</td><td>
We have provided further detail on the theoretical frameworks used to support the development of the intervention. In addition, where specific models and frameworks were used for different components of the intervention, this has also been detailed.

Section Intervention components: ‘The ELLY study adopted a community-based participatory research approach [28], where community members were active and engaged at all stages of the research process. It was co-designed by two disadvantaged communities for use in disadvantaged communities. Development of the ELLY intervention was informed by guidance on development and evaluation of complex interventions (MRC/UKRI Guidance on complex interventions) [29]. The framework by Adams et al (2014) [30] was used to identify all domains of the incentive scheme for which choices needed to be made. The behavioural theory of ELLY was informed by the COM-B model [31]. The intervention is described using the Template for Intervention Description and Replication (TIDieR) Checklist[32] a summary of which is provided in Figure 2.’

Figure 2: TIDieR checklist for ELLY
</td></tr>
<tr><td>No strengths or weakness are presented</td><td>
Section ‘Discussion’ has now been divided into subsections ‘Principal findings’, ‘Strengths and weaknesses’ and ‘Relation to other studies’. In addition, further detail has been added to the ‘Strengths and weaknesses’ section: ‘The ELLY study was effective in producing a co-designed intervention with two disadvantaged communities for use in disadvantaged communities. The intervention is underpinned by systematic review findings, theory informed and extends the evidence for use of financial incentive interventions for supporting healthy weight and wellbeing in disadvantaged communities. [49] The progression criteria set by an independent study steering committee were sufficiently met to proceed to a full trial.
The feasibility study was not powered to detect effects on positive weight change or improved wellbeing, therefore findings should be interpreted with caution. Possible expectation effects, the short study
</td></tr>
</table>

	time frame, and assumptions of directionality of relationships were present in this research and should be addressed in its extensions. Figures provided relating to attendance at weekly soups and questionnaire data are reliant on participant self-reporting. The mean average soup cost per person of £12.02 over the 12 weeks is calculated from the cost of soup ingredients and does not account for wider opportunity costs (e.g. time taken to prepare soup, electricity costs, cost of volunteering). Although communities were chosen for their disparate nature, further consideration should be given to the mix and diversity of communities in a future evaluation to maximise generalisability of findings and contribution to theory and intervention development. Careful consideration of what costs should be included in cost-effectiveness calculations, aligned to the perspective taken (e.g. consideration of societal costs, public-sector costs) should be given for future evaluation of the intervention.'
**Reviewer 2 comments (Dr. Dimitrios Koutoukidis, University of Oxford)**	
Comments	Response
Thank you for the opportunity to review this interesting and well-written paper. It is a well conducted feasibility trial with a rounded approach to process evaluation. I think the results will add to the evidence base. A few comments below for consideration.	Thank you to the reviewer for their comments on how well the feasibility trial was conducted and the quality of the paper.<br><br>We appreciate your helpful comments and suggestions and have addressed these below.
**Abstract**<br>Intervention: unclear what "unconditional soup" and "assets" are, suggest reword the whole section for clarity.	Further detail has been added to the intervention description to clarify what the soup and assets components of ELLY are. Section Abstract/Intervention: Intervention: 'The Enjoy Life LocallY (ELLY) intervention comprised free soup twice weekly (café/delivery/pickup); loyalty card stamped for engagement in community assets (such as local activities, groups and clubs) exchanged for a £25 shopping card when a participant attends a minimum of 9 assets over 12 weeks; goal-setting; information resources; self-monitoring of weight and wellbeing.'<br><br>In addition, we have added a further figure to the manuscript in the Intervention Components section. Figure 2 provides the TIDieR checklist of the ELLY intervention which provides further clarity around the main components.
**Abstract**<br>Conclusion not supported by results section - no data on weight or well being in the results section. I	We have added data relating to weight change to the Results section (Table 4) of the main paper:<br><br><table><tr><th></th><th>Mean</th><th>SD</th><th>95% CI</th></tr><tr><td>Weight change (kg), mean (SD)</td><td>-0.43</td><td>3.33</td><td>-1.26, 0.40</td></tr><tr><td>Weight change (%), mean (SD)</td><td>-0.35</td><td>3.68</td><td>-1.26, 0.56</td></tr></table>

<table>
<tr>
<td>suggest this is not overstated.</td>
<td>
<table>
<tr><td>Body Mass Index (kg/m^2)</td><td>-0.15</td><td>1.26</td><td>-0.44, 0.14</td></tr>
<tr><td>EQ-5D-5L index score</td><td>0.02</td><td>0.20</td><td>-0.26, 0.07</td></tr>
<tr><td>WEMWBS</td><td>0.80</td><td>9.74</td><td>-1.44, 3.04</td></tr>
<tr><td>Social connectedness scale</td><td>0.80</td><td>14.6</td><td>-2.56, 4.16</td></tr>
</table>

We have re-worded our conclusions in the abstract to focus soly on primary outcomes of the study. Abstract/Conclusion: ‘Conclusions: The ELLY study recruited and retained participants from two disadvantaged communities in Scotland. The study was acceptable to participants and feasible to deliver. A full trial is warranted to determine effectiveness and cost-effectiveness, with consideration of scalability.’

We have also clarified that change in outcomes prioritised by communities for a future trial (secondary outcomes) are not powered to detect statistical significance.
Article summary/Strengths and weaknesses of the study: ‘The feasibility study was not powered to detect effects on weight-related or wellbeing outcomes and change in outcome measures should be interpreted with caution.’
</td>
</tr>
<tr>
<td>“Goal setting options (personal, weight, wellbeing goals) discussed with participants at baseline;” this needs more clarity - how long was the baseline counselling, did it involve anything beyond goal setting, what does “discussed” mean (were they explicitly asked to set goals or given simply the option to)?. What goals were these? goals for behaviours, for behavioural outcomes or both? How specific was it? Was it accompanied by an action plan?</td>
<td>More specific information regarding the goal setting activity has been provided including how goals were set and the parameters in which participants were supported to do this.
Section Methods/Intervention components:

Information relating to goal setting, has been added to the following sections: time taken to discuss goal Methods/‘Baseline appointment’: ‘The topic of goal setting (rationale and how it can be helpful) had already been introduced to participants in the ELLY Participant Information Sheet. In the baseline appointment, the researcher and participant engaged in discussion around potential goals the participant may wish to set. The mean average time taken for baseline appointments was 45 minutes, with questionnaire completion taking an average 20 minutes, and goal setting discussions, taking an average of 10 minutes.

Intervention Components: ‘…the option to set goals. Goal setting options were discussed at the baseline appointment, where participants were informed about the optional aspect of goal setting for ‘living well’. Participants were given the opportunity to set (outcome or behaviour) goals under the topics of personal, weight and wellbeing. Goal setting was participant driven however the researcher encouraged generation of SMART (Specific, Measurable, Achievable, Relevant, and Time-bound) goals to achieve over the 12 weeks. No specific action plans were developed however the researcher signposted the participants to the other intervention components and community assets. Goals set were reviewed at 12-week appointments;’</td>
</tr>
<tr>
<td>I can see how nutrition-related groups and PA are the expected choices of</td>
<td>Community feedback indicated that assets should be broad and inclusive, rather than targeted. Community citizens wanted autonomy around what assets they engaged in and therefore the ELLY intervention was not prescriptive in this respect.</td>
</tr>
</table>

groups to help with healthy weight - but what about arts and crafts and social groups - how are these linked with the intervention aims? One aspect is wellbeing but did they also contribute to healthy weight?	Further detail has been provided to Section Methods/Intervention Components to provide clarity around the premise for wide-ranging assets: 'The ELLY intervention is a place-based, asset-based incentive system. Community consultation indicated that an intervention focusing solely on weight was felt stigmatising and not inclusive of all community citizens. Citizens expressed a desire for an intervention to support them as a "whole person" (recognising mental, physical, social, spiritual aspects), rather than a focus on one component alone. The resulting intervention adopts a holistic approach to supporting healthy weight and wellbeing, acting as a connector to existing assets and promoting autonomy. The intervention is not prescriptive in which ELLY assets participants should engage in, or exclusive in incorporating only assets seen to be directly supportive of healthy weight and/or wellbeing (for example, a walking club). Assets such as a writing group or craft club (two ELLY assets in C2), which may have indirect benefits to healthy weight and wellbeing, such as providing friendship, reducing social isolation and providing an opportunity for physical activity, were included.'
It would be useful to present data on Table 3 split by those with weight loss goals, personal goals, and wellbeing goals in the supplement, So that we can see if eg those setting weight loss goals found the soup provision more helpful.	We cross-tabulated type of goal set with engagement in different aspects of ELLY, however, as the percentage of participants setting goals across all three categories (weight, wellbeing and personal) was high (85%, 88% and 87% respectively) the numbers of those not setting particular goals were small. In addition, there was little/no behaviour/acceptability differences between the goal-setters and those that didn't set a particular goal. For example, 10/75 did not set a weight goal, but 50% of these engaged in the soup cafes.<br><br>We have added information on this analysis and findings to Section Results/Goal Setting: 'Analysis of goals set and engagement in other ELLY components was conducted to determine if setting particular goals led to a greater likelihood of engagement in different components. For example, did participants who set a weight goal engage more with the soup cafés that those who did not? Findings suggest there was no significant difference in engagement of different ELLY components between goal-setters and non-goal setters. It should be noted that numbers of participants choosing not to set particular goals was low, so this finding is based on small numbers.'
Indicative effects on healthy weight and wellbeing at 12-weeks: I suggest the authors reword this section by simply providing the estimates descriptively but not claiming potential/promising	We agree that use of the term indicative effects is misleading. We have removed this and instead referred to 'change' of measures. In addition, we have added weight change data to complement the BMI data provided.<br><br>Effects on weight-related and wellbeing outcomes at 12-weeks: 'Small changes are evident in all outcomes collected (Table 4).'<br><br>Revised Table 4 (see above)

effects, as the 95% CIs are crossing 0. I would replace BMI with weight change. It would be useful to specify in the stats section the method with which the CIs were calculated. It is unclear how missing data were handled for weight.	A sentence has been added to indicate the method for calculating confidence intervals (Section Methods/Analysis/Quantitative Analysis: 'Confidence intervals for proportions were calculated by the study statistician and derived using the normal approximation and for means using the standard normal distribution.'<br>A sentence has been added to indicate how missing data were handled for weight (Section Methods/Analysis/Quantitative Analysis: 'Missing data was handled by following the appropriate guidelines for each scale, with the exception of the Social Connectedness Scale – Revised, where in the absence of guidelines, we applied an adaption of the WEMWBS guidelines as used by Phillips et al 2019 [47]. For weight-related outcomes observed data only was included.'
Page 22 line 32: suggest replace conditions with aspects - weight or behaviours are not conditions.	Agreed that 'aspects' feels a more appropriate word to use. Thank you for the suggestion. Manuscript has been updated: 'A holistic approach to health wellbeing, rather than a focus on individual, potentially stigmatising aspects like weight or behaviour was shown in this study to be preferred by communities.'
Conclusion: "The design of a full scale evaluation requires careful consideration to ensure its appropriateness in addressing study objectives":this is fair but there are no lessons learnt from the feasibility study in the discussion at the moment. It would be good to elaborate on those in the discussion.	Section Discussion has now been divided into subsections Principal findings, Strengths and weaknesses and Relation to other studies. In addition, further detail of considerations for future studies has been added to the Conclusion: 'This study demonstrates the feasibility of co-designing and implementing a novel community-based, incentive intervention to support healthy weight and wellbeing. A larger study is warranted to determine effectiveness and cost-effectiveness, with consideration of scalability. The design of a full scale evaluation requires careful consideration to ensure its appropriateness in addressing study objectives. Community-based intervention studies can produce methodological challenges: how best to cluster across communities, how to ensure contextual differences are accounted for and how to ensuring a one-size-fits-all intervention is flexible enough to address local needs, whilst maintaining fidelity. In the ELLY study, outcome measured prioritised by communities were multiple and of equal importance, necessitating discussion around use of co-primary outcomes in a future study. In all decisions around study design of a full scale evaluation, ensuring equitable engagement of community citizens will be crucial in maximising study success.'
Typos<br>Page 5 line 37: weight mentioned twice<br>Page 22 line 31: health and wellbeing<br>Spaces before commas in a few places	Thank you for highlighting these. The typos have now been corrected.

VERSION 2 - REVIEW

**Reviewer**	**2**
**Name**	**Koutoukidis, Dimitrios**
**Affiliation**	**University of Oxford, Nuffield Department of Primary Care Health Sciences**
**Date**	**06-Jan-2025**
**COI**	

I am happy with the authors' response. Two minor points

- It would be helpful to specifically mention somewhere in the manuscript that the trial was not pre-registered (rather than just as NA in the CONSORT checklist - it should not be an NA as this is an applicable point as all trials should be registered).

- Delete the sentence "small changes were evident" as this still implies a significant difference - but the study shows no evidence of an effect.

VERSION 2 - AUTHOR RESPONSE

**Reviewer 2 comments (Dr. Dimitrios Koutoukidis, University of Oxford)**	
Comments	Response
- It would be helpful to specifically mention somewhere in the manuscript that the trial was not pre-registered (rather than just as NA in the CONSORT checklist - it should not be an NA as this is an applicable point as all trials should be registered).	We have included a section 'Trial Registration' where we detail that the ELLY feasibility trial was not pre-registered (page 25 of Main Document):<br>**Trial registration**<br>The ELLY feasibility study was not pre-registered.<br>We have also updated the CONSORT checklist item 23, replacing NA with page 25 to indicate trial registration information is now part of the main document.
- Delete the sentence "small changes were evident" as this still implies a significant difference - but the study shows no evidence of an effect.	We have removed this sentence from Section '**Effects on weight-related and wellbeing outcomes at 12-weeks'** and replaced it with:<br>The effects on outcomes collected are shown in Table 4.
